# Delivery of oligonucleotide‐based therapeutics: challenges and opportunities

**DOI:** 10.15252/emmm.202013243

**Published:** 2021-04-06

**Authors:** Suzan M Hammond, Annemieke Aartsma‐Rus, Sandra Alves, Sven E Borgos, Ronald A M Buijsen, Rob W J Collin, Giuseppina Covello, Michela A Denti, Lourdes R Desviat, Lucía Echevarría, Camilla Foged, Gisela Gaina, Alejandro Garanto, Aurelie T Goyenvalle, Magdalena Guzowska, Irina Holodnuka, David R Jones, Sabine Krause, Taavi Lehto, Marisol Montolio, Willeke Van Roon‐Mom, Virginia Arechavala‐Gomeza

**Affiliations:** ^1^ Department of Paediatrics University of Oxford Oxford UK; ^2^ Department of Human Genetics Leiden University Medical Center Leiden The Netherlands; ^3^ Department of Human Genetics, Research and Development Unit National Health Institute Doutor Ricardo Jorge Porto Portugal; ^4^ Department of Biotechnology and Nanomedicine SINTEF AS Trondheim Norway; ^5^ Department of Human Genetics and Donders Institute for Brain, Cognition and Behaviour Radboud University Medical Center Nijmegen The Netherlands; ^6^ Department of Biology University of Padova Padova Italy; ^7^ Department of Cellular, Computational and Integrative Biology ‐ CIBIO University of Trento Trento Italy; ^8^ Centro de Biología Molecular Severo Ochoa UAM‐CSIC CIBERER, IdiPaz Universidad Autónoma de Madrid Madrid Spain; ^9^ SQY Therapeutics Montigny‐le‐Bretonneux France; ^10^ Department of Pharmacy Faculty of Health and Medical Sciences University of Copenhagen Copenhagen Ø Denmark; ^11^ Victor Babes National Institute of Pathology Bucharest Romania; ^12^ Department of Biochemistry and Molecular Biology University of Bucharest Bucharest Romania; ^13^ Department of Pediatrics Radboud University Medical Center Nijmegen The Netherlands; ^14^ Université Paris‐Saclay, UVSQ, Inserm, END‐ICAP Versailles France; ^15^ Department of Physiological Sciences Faculty of Veterinary Medicine Warsaw University of Life Sciences – SGGW Warsaw Poland; ^16^ Institute of Microbiology and Virology Riga Stradins University Riga Latvia; ^17^ MHRA 10 South Colonnade London UK; ^18^ Department of Neurology Friedrich‐Baur‐Institute Ludwig‐Maximilians‐University of Munich Munich Germany; ^19^ Institute of Technology University of Tartu Tartu Estonia; ^20^ Division of Biomolecular and Cellular Medicine Department of Laboratory Medicine Karolinska Institutet Huddinge Sweden; ^21^ Duchenne Parent Project España Madrid Spain; ^22^ Department of Cell Biology, Fisiology and Immunology Faculty of Biology University of Barcelona Barcelona Spain; ^23^ Neuromuscular Disorders Group Biocruces Bizkaia Health Research Institute Barakaldo Spain; ^24^ Ikerbasque, Basque Foundation for Science Bilbao Spain

**Keywords:** delivery, oligonucleotides, preclinical models, RNA therapeutics, safety, Synthetic Biology & Biotechnology, Chemical Biology, RNA Biology

## Abstract

Nucleic acid‐based therapeutics that regulate gene expression have been developed towards clinical use at a steady pace for several decades, but in recent years the field has been accelerating. To date, there are 11 marketed products based on antisense oligonucleotides, aptamers and small interfering RNAs, and many others are in the pipeline for both academia and industry. A major technology trigger for this development has been progress in oligonucleotide chemistry to improve the drug properties and reduce cost of goods, but the main hurdle for the application to a wider range of disorders is delivery to target tissues. The adoption of delivery technologies, such as conjugates or nanoparticles, has been a game changer for many therapeutic indications, but many others are still awaiting their eureka moment. Here, we cover the variety of methods developed to deliver nucleic acid‐based therapeutics across biological barriers and the model systems used to test them. We discuss important safety considerations and regulatory requirements for synthetic oligonucleotide chemistries and the hurdles for translating laboratory breakthroughs to the clinic. Recent advances in the delivery of nucleic acid‐based therapeutics and in the development of model systems, as well as safety considerations and regulatory requirements for synthetic oligonucleotide chemistries are discussed in this review on oligonucleotide‐based therapeutics.

## Graphical abstract

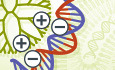

**Glossary**
Anti‐drug antibodies (ADAs): Antibody‐mediated immunogenicity elicited *in vivo* to a given drug. Drug‐specific antibodies can reduce the efficacy of the treatment and even fully inactivate the drug, and/or they can induce adverse effects.
Antisense oligonucleotides (ASOs): Single‐stranded oligonucleotides complementary to RNA target sequences.
Aptamers: Single‐stranded oligonucleotides (20‐100 nucleotides) which adopt three‐dimensional structures that allow them to bind very specifically to protein target sites.
Blood–brain barrier (BBB) and blood–spinal cord barrier (BSCB): Selectively permeable membranes of the central nervous system (CNS) vasculature. Only small molecules (molecular weight below 400‐500 Da) and high lipid solubility (logP value of approximately 2.1) can cross these vascular barriers. Generally, oligonucleotides display a molecular weight of approximately 10 kDa and are hydrophilic; hence, they are too large and hydrophilic to cross biological barriers by passive diffusion.
Cell‐penetrating peptides (CPPs): Short cationic and/or amphipathic peptides (usually less than 30 amino acids) capable of translocating different types of cargoes across biological barriers and cell membranes. CPPs can be directly conjugated to oligonucleotides (ONs) or used to encapsulate ONs into nanoparticles.
European Medicines Agency (EMA): Agency of the European Union in charge of the evaluation and supervision of medicinal products. The EMA facilitates development and access to medicines, evaluates applications for marketing authorisation and monitors the safety of human and veterinary medicines.
Food and Drug Administration (FDA): The federal agency of the United States Department of Health and Human Services, responsible for protecting public health by ensuring the safety, efficacy and security of human and veterinary drugs.
Gapmer: Chimeric antisense oligonucleotides (ASOs) that contain a central block of DNA nucleotides, flanked by modified sequences, usually containing 2′‐O‐modified or locked nucleic acid (LNA) chemistries. Gapmers are used for gene silencing by stimulating RNA cleavage through the recruitment of RNase H.
Lipid nanoparticles (LNPs): Delivery systems based on LNPs are composed of one or several lipid components, often an ionisable cationic lipid used for complexation of polyanionic DNA/RNA and stabilising helper lipids such as distearoylphosphatidylcholine (DSPC) and cholesterol. In addition, LNPs may be stabilised sterically by surface coating with polyethylene glycol (PEG). LNPs have a complex internal lipid architecture that is well suited for stable and efficient encapsulation of DNA/RNA cargoes.
MicroRNAs (miRNAs): Small noncoding RNAs (∼22 nt), which regulate gene expression at the post‐transcriptional level by degrading target mRNAs, when complementary to the sequence, or inhibiting their translation when not fully complementary. Each miRNA can influence the expression of hundreds of mRNAs.
Pharmacodynamics (PD): The relationship between the drug concentration at the site of action and the observed biochemical response and its efficacy.
Pharmacokinetics (PK): The time course of drug absorption, distribution, metabolism, excretion and toxicity (ADMET), as well as the liberation of a drug from its formulation.
Phosphorodiamidate morpholino oligonucleotides (PMOs): Oligonucleotides containing uncharged chemistry. The nucleic acid backbone has been replaced with 6‐membered morpholino rings and phosphorodiamidate linkages, while retaining standard nucleobases.
Peptide nucleic acid (PNA): Uncharged oligonucleotide chemistry with amide bond linkages between the nucleobases. PNAs are manufactured by peptide synthesis.
RNAse H cleavage: RNAse H hydrolyses the phosphodiester bonds of RNA when hybridised to DNA.
Small interfering RNA (siRNA): Double‐stranded RNA (~21 nt) composed of a guide strand complementary to the target mRNA and a passenger strand. siRNAs act within the endogenous RNA‐induced silencing complex (RISC) to degrade mRNA.
Toll‐like receptors (TLRs): Pattern‐recognition receptors usually found on the plasma or endosomal membranes of sentinel cells such as macrophages and dendritic cells (DCs). Activation of TLRs can promote an inflammatory response. For example, TLR9 is activated by unmethylated cytidine‐phosphate‐guanosine (CpG) dinucleotides present in bacterial and viral DNA.

## Introduction

Synthetic oligonucleotides (ONs) are small, single‐ or double‐stranded pieces of modified nucleic acids that have been exploited as therapeutic modalities in different ways (Table 1). The unique characteristic of ONs is that they bind to their target via Watson–Crick base pairing, enabling intervention at a genetic level by targeting RNA in a specific manner (Zamecnik & Stephenson, 1978). ONs encompass many types of nucleic acid‐based therapeutics, including antisense oligonucleotides (ASOs), small interfering RNA (siRNA), anti‐miRNA (antagomirs), miRNA mimics (agomirs), aptamers and unmethylated CpG‐containing ONs. Depending on their mechanism of action, treatment with therapeutic nucleic acids may cause decreased, increased or restored protein expression. Currently, 11 ON‐based drugs across many disease areas have received regulatory approval by the US Food and Drug Administration (FDA), the European Medicines Agency (EMA) and/or the Japanese Ministry of Health, Labour and Welfare. However, further therapeutic development is challenged by unfavourable absorption, distribution, metabolism, excretion and toxicity (ADMET) properties for most clinical applications (Godfrey *et al,* 2017). This review mainly focuses on the development of single‐stranded ONs and covers (i) the numerous methods developed to date to deliver ONs across biological barriers, (ii) the model systems used to test ONs and (iii) the hurdles existing for translating laboratory breakthroughs to the clinic. The content represents the joint efforts of members of the EU Cooperation of Science and Technology (COST) network *Delivery of RNA Therapeutics* (DARTER, COST Action 17103, www.antisenserna.eu), which aims to facilitate RNA‐targeting nucleic acid‐based drugs to reach their full potential.

**Table 1:** Mechanisms of action of therapeutic oligonucleotides.

Modality	Mechanism	Example(s)
RNase H	RNase H‐mediated cleavage of target transcript	Gapmers
Steric Blockage	Interference with post‐transcriptional RNA‐binding elements, *e.g*. splicing modulation and blocking endogenous miRNA	2nd and 3rd generation ASOs and antagomirs
Protein Binding	Bind target proteins in a structure‐specific manner	Aptamer
Innate Immunity	Inhibits protein expression via target‐specific mRNA degradation	Unmethylated CpG‐containing ONs
RNAi	Inhibition of gene expression via target‐specific mRNA degradation	siRNAs, microRNAs

## Chemistry dictates the drug properties of oligonucleotides

**Figure 1:**
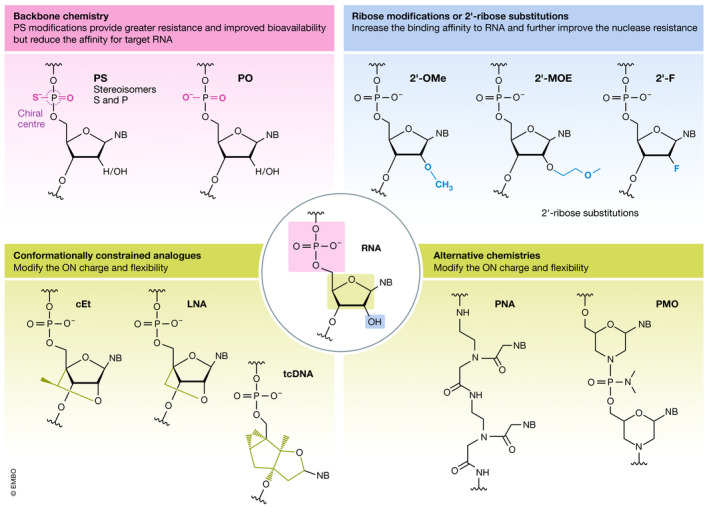
Oligonucleotide chemistries Commonly used nucleic acid chemistries. The often used phophorothioate (PS) backbone replaces the natural phosphodiester (PO). Modifications to the ribose at the 2ʹ‐*O* position of RNA and 2ʹ‐position of DNA include the 2ʹ‐*O*‐methyl (2ʹ‐*O*Me), 2ʹ‐*O*‐methoxy‐ethyl (2ʹ‐MOE) and 2ʹ‐fluoro (2ʹ‐F) are the most commonly used. Conformationally constrained DNA analogues, locked nucleic acid (LNA), constrained 2′‐O‐ethyl (cEt) and tricyclo‐DNA (tcDNA), provide greater binding affinity. LNA and cEt are constrained by a methyl bridged from the 2′‐O and 4′ position of the ribose. tcDNA introduces of an ethylene bridge with a cyclopropane ring between the 3′ and 5′ carbon positions of ribose. Alternative chemistries include changes in the nucleobase, *e.g*. phosphorodiamidate morpholino oligomers (PMO) and peptide nucleic acid (PNA).

Therapeutic nucleic acids are chemically modified in several ways to endow them with properties such as increased resistance to nucleases and improved target binding affinity (Jarver *et al,* 2014) (Fig 1). Each modification confers the ON different properties, and some may be combined, but other modifications are not compatible or may modify the ON in ways that complicate their synthesis or interfere with the mechanisms by which they exert their effect. First‐generation chemistries include the widely used phosphate backbone modifications, *e.g*. phosphorothioate (PS), which imparts resistance to endonucleases and improves bioavailability by reducing renal clearance due to increased affinity for serum proteins (Eckstein, 2014). However, this modification also reduces the affinity for the target RNA. Second‐generation chemistries include ribose modifications at the 2′‐*O* position of RNA and 2′ position of DNA, of which the 2ʹ‐*O*‐methyl (2ʹ‐*O*Me), 2ʹ‐*O*‐methoxy‐ethyl (2ʹ‐MOE) and 2ʹ‐fluoro (2ʹ‐F) modifications are the most commonly used types. These modifications increase the binding affinity to RNA and further improve the nuclease resistance. An even greater binding affinity chemistry is the conformationally constrained DNA analogues locked nucleic acid (LNA) and tricyclo‐DNA (tcDNA). LNA contains a methyl bridge between the 2′‐*O* and 4′ position of the ribose ring (Koshkin *et al,* 1998; Obika *et al,* 1998). The backbone considerably changed for tcDNA via introduction of an ethylene bridge with a cyclopropane ring between the ribose 3' and 5' carbon positions (Renneberg & Leumann, 2002). The bridge imposes a locked conformation on the ribose ring, which is ideal for binding to RNA. All first‐ and second‐generation chemistries are compatible with nucleic acid synthesis and can easily be mixed with DNA and RNA in ON chimeras. Third‐generation chemistries include changes in the nucleobase, *e.g*. phosphorodiamidate morpholino oligomers (PMO) (Summerton & Weller, 1997) and peptide nucleic acid (PNA) (Nielsen *et al,* 1991; Hanvey *et al,* 1992). For PMOs, the nucleic acid backbone has been replaced with a 6‐membered morpholino ring and phosphorodiamidate linkages, while retaining standard nucleobases. The nucleobases of PNAs are linked by amide bonds, which are synthesised similarly to peptides. Both PMO and PNA are uncharged, very resistant to nucleases, and display variable affinity for the target RNA (Smulevitch *et al,* 1996; Summerton & Weller, 1997). The choice of chemical modifications is largely dictated by the modality and the target tissue.

**Figure 2:**
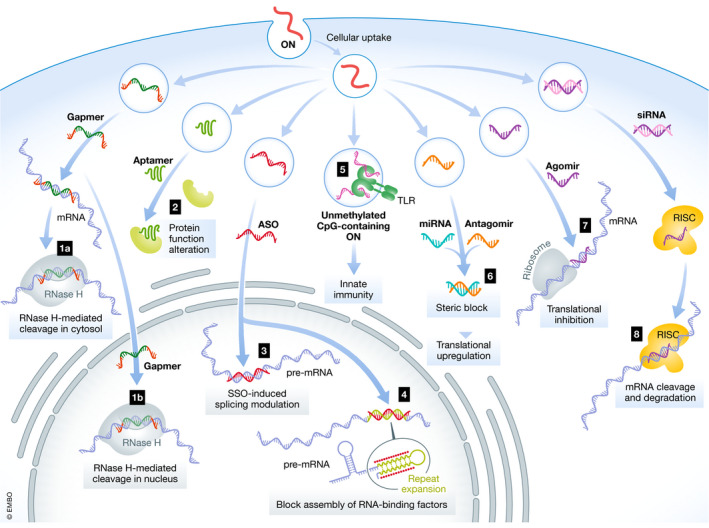
Mechanisms and location of action for oligonucleotides Representative mechanisms of action and intracellular localisation for (1) gapmer and mRNA degradation, (2) aptamer, (3) nuclear steric blockage for splice switching, (4) blockage the assembly of RNA‐binding factors, (5) TLR activation of innate immunity, (6) miRNA and antagomir, steric block, translational upregulation, (7) agomir, translational inhibition, and (8) siRNA, RISC, RNAi silencing ONs.

Single‐stranded ASOs complementary to target RNA were first utilised therapeutically by exploiting RNase H cleavage of DNA/RNA hybrids (Stein & Hausen, 1969; Wu *et al*, 2004) (Fig 2). RNase H‐inducible ASOs are designed as gapmers, where central DNA nucleotides are flanked by RNase H‐resistant modified nucleotides (Wahlestedt *et al,* 2000). The modified sequences improve target affinity while the central DNA sequence forms the DNA/RNA hybrid for RNase H recognition and cleavage (Monia *et al,* 1993). Fully modified second‐ and third‐generation ASO chemistries act through RNase H‐independent mechanisms (Fig 2) (Jarver *et al,* 2014). Steric blocking ASOs can inhibit or activate translation through the binding to regulatory elements*, e*.*g*. upstream open reading frames (Liang *et al,* 2016b; Liang *et al,* 2017). A common therapeutic modality is the modulation of pre‐mRNA splicing (Arechavala‐Gomeza *et al,* 2014), which is used to induce or suppress exon inclusion. In Duchenne muscular dystrophy (DMD) patients, ASO‐induced exon skipping of mutated dystrophin pre‐mRNA restores the reading frame and allows for the production of partially functional, rather than non‐functional, dystrophin protein (Mitrpant *et al,* 2009). In contrast, for spinal muscular atrophy (SMA) patients, ASOs increase the level of exon 7 inclusion in survival motor neuron 2 (*SMN2*) mRNA, leading to increased levels of SMN protein (Singh *et al,* 2006). Similarly, ASOs can also induce the skipping of pseudoexons (Collin *et al,* 2012) or block RNA‐splicing factors from recognising cryptic splice sites (Rivera‐Barahona *et al,* 2015). ASOs can also sterically block the union of RNA‐binding factors to repeat expansion regions of pathogenic mRNAs (Fig 2). In myotonic dystrophy 1, expanded microsatellite repeats sequester RNA‐binding factors within nuclear expansion RNA foci (Miller *et al,* 2000). ASOs targeting the CUG repeat expansion mRNA release the sequestered RNA‐binding factors and reverse the phenotype (Klein *et al,* 2019). RNA interference (RNAi)‐based therapies, *i.e*. double‐stranded siRNA and single‐stranded microRNA (miRNA), exploit the endogenous RNAi pathway in the cytosol (Fire *et al,* 1998) to silence or modulate the expression of specific proteins (Fig 2). Commonly used chemical modifications for siRNA, including 2ʹ‐OMe and 2ʹ‐F modifications, decrease RNase recognition and are well tolerated throughout the entire siRNA duplex (Watts *et al,* 2008). In addition, these modifications are widely used to decrease immune stimulation (Judge *et al,* 2006). ASOs can influence miRNA function, either by sequestering a miRNA (antagomir) or by generating a miRNA mimic (agomir). Notably, a single miRNA generally regulates the expression of multiple genes in a given pathway; hence, antagomirs and agomirs have the potential to mediate increased or decreased expression of multiple genes, respectively (Friedman *et al,* 2009). Finally, two types of ONs which do not work through Watson–Crick base pairing are aptamers and unmethylated CpG‐containing ONs. Aptamers are single‐stranded ONs (20–100 nucleotides) selected from randomised libraries based on their high‐avidity binding to specific targets (Ellington & Szostak, 1990; Tuerk & Gold, 1990). They adopt three‐dimensional structures that bind to protein target sites through attractive electrostatic interactions and pocket‐like structures (Ellington & Szostak, 1990), and they display binding affinities to their receptor targets which are comparable to those of monoclonal antibodies (Jayasena, 1999). Unmethylated CpG‐containing ONs include a cytosine‐guanine motif coupled with a phosphodiester (PO) or PS backbone. Unmethylated CpG motifs are commonly found in bacterial DNA and activate the immune system through Toll‐like receptor 9 (TLR9). Unmethylated CpG‐containing ONs have been tested clinically as vaccine adjuvants and for cancer immunotherapy (Krieg & Davis, 2001; Krieg, 2006, 2007).

## Delivery systems for oligonucleotides

**Figure 3:**
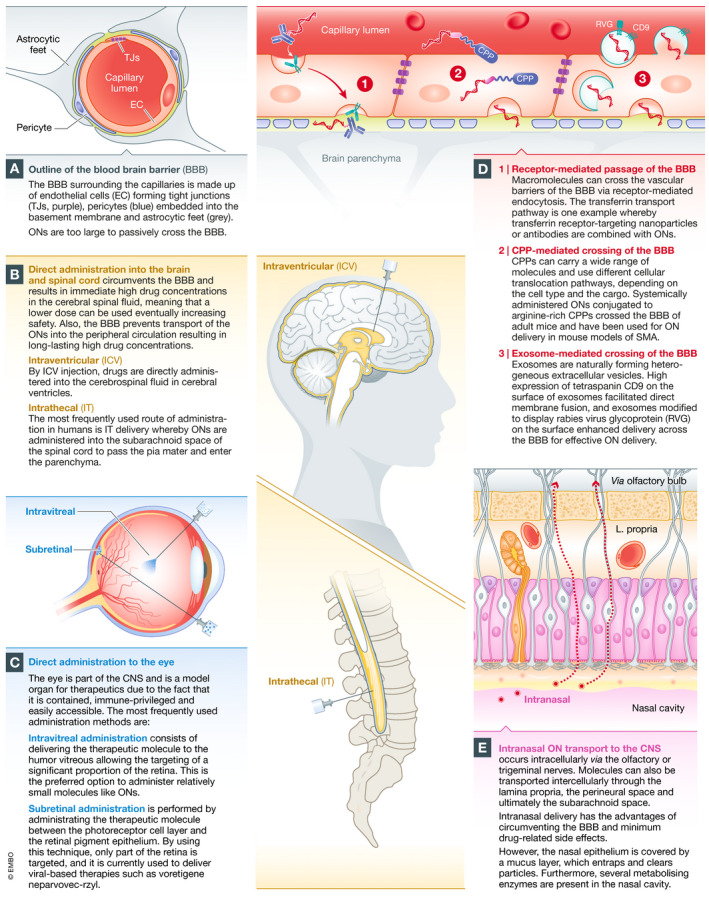
Delivery of oligonucleotides to the brain and eye (A) ONs are prevented from passive diffusion into the central nervous system (CNS) by the vascular BBB. (B) ONs without a delivery reagent require direct administration into the brain or spinal cord. The most frequently used CNS administration route in humans is intrathecal (IT) administration, where ONs are administered into the subarachnoid space of the spinal cord to pass the pia mater and enter the parenchyma. This results in an immediate high ON concentration in the cerebral spinal fluid, meaning that a lower dose can be used, which reduces side effects. Also, the BBB prevents transport of ONs into the peripheral circulation resulting in long‐lasting high ON concentrations. (C) The eye is a contained and immune‐privileged organ of the CNS that allows local delivery. ONs are effective and well tolerated when administered directly by intravitreal injection. Subretinal delivery is also possible, but the treated area will be reduced. (D) Certain macromolecules can cross the vascular barriers via receptor‐mediated endocytosis after systemic administration (Pardridge, 2007). The transferrin transport pathway has been exploited in several rodent studies to carry ONs into the brain parenchyma (Lee *et al,* 2002; Kozlu *et al,* 2014). Systemically delivered ONs covalently conjugated to arginine‐rich CPPs have been shown to cross the BBB in mice (Du *et al,* 2011) and have been used for ON delivery in mouse models of SMA (Hammond *et al,* 2016). Several studies have shown exosome‐mediated delivery of small RNAs across the vascular barriers into the CNS (Alvarez‐Erviti *et al,* 2011; Yang *et al,* 2017). (E) Drugs dosed by intranasal administration can be transported into the brain along the olfactory, trigeminal nerve and rostral migratory stream (Curtis *et al,* 2007).

**Figure 4:**
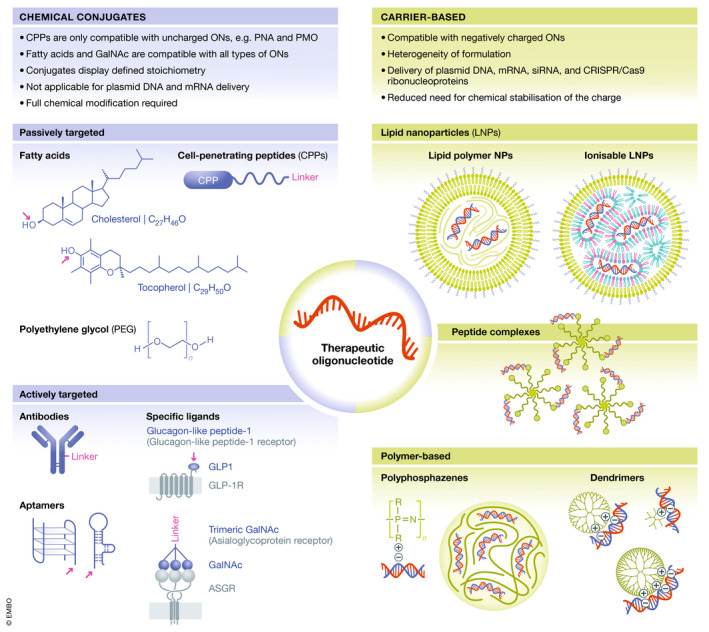
Delivery technologies for oligonucleotides Delivery technologies used to improve the ADMET properties of ONs, including chemical conjugates (left) and nanoparticulate carriers (right). Polymers, cell‐penetrating peptides (CPPs) and lipids represent examples of molecules used for covalent conjugation to ONs for passive targeting, whereas covalent conjugation of ONs to antibodies, receptor ligands and aptamers are applied for active targeting. Drug conjugates display a defined stoichiometry. CPP conjugation is only compatible with uncharged ONs, *e.g*. PMOs and PNAs, whereas lipids and GalNAc are compatible with all types of ONs. Nanoparticulate carriers can be used to encapsulate negatively charged ONs and can be based on lipids, *e.g*. lipid nanoparticles (LNPs) and exosomes, polymers, *e.g*. dendrimers, poly(lactide‐co‐glycolic acid) (PLGA) and polyphosphazenes, and peptides, or on hybrid systems composed of several different types of compounds. The complexity of these systems poses new challenges in the development with respect to cost, manufacturability, safety, quality assurance and quality control.

The sites of action for ONs lay within the intracellular space. Consequently, they need to overcome several biological barriers to reach their pharmacological targets *in vivo*. PS‐modified ONs bind reversibly to plasma proteins, *e.g*. albumin, which increases their plasma half‐life and facilitates their distribution and accumulation in the liver, kidneys, spleen, lymph nodes and bone marrow (Geary, 2009). Targeting tissues beyond these organs has had clinical success for local delivery to the eye, brain and spinal cord via intravitreal (IVT) and intrathecal (IT) administration, respectively (Hache *et al,* 2016; Cideciyan *et al,* 2019) (Fig 3). Both routes bypass renal clearance and maintain high ON exposure to the cellular microenvironment for efficient uptake. Additionally, significant advances for pulmonary delivery of RNA therapies have been extensively reviewed elsewhere (Chow *et al,* 2020; Shaffer, 2020). However, systemic administration of ONs has been less successful due to poor tissue uptake. Cellular uptake of ONs occurs predominantly via different types of endocytosis. ONs are subsequently trafficked into the endolysosomal system, from where they need to escape to avoid degradation in the lysosomal environment (Crooke *et al,* 2017). Only a very small ON dose fraction escapes the endosomes and becomes available at the site of action (Gilleron *et al,* 2013). Single‐stranded ONs, such as PS ASOs, which are relatively small, uncharged and/or hydrophobic, can productively enter cells and escape the endosomes into the cytoplasm and nucleus without the need for a delivery agent (Liang *et al,* 2016a) in a process referred to as gymnosis (Stein *et al,* 2010), but relatively high ON doses are required for this process to take place. However, most of RNA‐based therapeutics, *e.g*. double‐stranded siRNA, are too large and charged to enter cells unassisted and require a delivery agent. Accelerating the rate of cellular uptake, intracellular trafficking and endosomal escape has been a driving force behind advances in many chemical modifications and delivery agents (Juliano *et al,* 2018; Biscans *et al,* 2020). A wide variety of delivery approaches improve the transport and bioavailability of ONs (Fig 4) (Roberts *et al,* 2020). These include (i) direct conjugation to carriers and (ii) incorporation into nanoparticulate carriers, both with the aim of improving the ADMET properties.

### Chemical conjugates

Chemical conjugation of molecules to therapeutic ONs is an attractive strategy for improving ADMET properties. As chemical conjugates, ONs are exposed to serum, and therefore, full chemical modification of ONs is needed to protect them from degradation. Polymers, peptides, lipids, receptor ligands and aptamers represent examples of molecules used for conjugation (Fig 4).

#### Polymers

Covalent conjugation of polyethylene glycol (PEG) improves the ADMET properties of drugs. PEGylation has been applied mainly for therapeutic proteins, but more recently also for ONs, *e.g*. the marketed aptamer‐PEG conjugate pegaptanib directed against vascular endothelial growth factor (VEGF) (Ng *et al,* 2006). PEG is a highly flexible, non‐charged and hydrophilic polymer with end groups available for functionalisation. PEG shields the conjugated drug cargo via formation of a hydration shell, which sterically blocks other biomacromolecules from binding to the drug. Also, PEGylation prolongs the circulation time by reducing renal excretion and increasing ON stability. The ADMET properties of PEGylated ONs are dependent on the physicochemical properties of the PEG moiety, including the molecular weight, the type of end group modification and the PEG architecture (linear or branched). For example, pegaptanib contains a 40 kDa Y‐shaped PEG, which causes the aptamer binding affinity to decrease fourfold compared with the parent aptamer, whereas the antiangiogenic efficacy is increased, which is attributed to prolonged tissue residence time due to increased half‐life (Ng *et al,* 2006).

#### Peptides

Cell‐penetrating peptides (CPPs) are short cationic and/or amphipathic peptides, usually less than 30 amino acids long, capable of translocating different types of cargoes across biological barriers and cell membranes (Foged & Nielsen, 2008; Pooga & Langel, 2015; Lehto *et al,* 2016). CPPs can be used as direct conjugates or to encapsulate oligonucleotides into nanoparticles, which is discussed further in the next section. Once inside the cells, CPPs may also improve endosomal escape (Cleal *et al,* 2013). However, the cationic charge often restricts their covalent conjugation to charge‐neutral ON chemistries (PNAs and PMOs) due to electrostatic interactions between anionic ONs and cationic CPPs that result in aggregation. For systemic diseases, CPP‐ONs circumvent cell‐specific receptors, allowing for pharmacological activity across multiple tissues, and they have been developed for uptake into particularly impervious tissues, *e.g*. skeletal muscle, heart and CNS (Hammond *et al,* 2016; Betts *et al,* 2019), as well as targeting viral and bacterial infections (Burrer *et al,* 2007; Geller *et al,* 2013; Geller *et al,* 2018). At the time of this review, a phase I clinical trial for safety and tolerability of an arginine‐rich CPP‐ASO conjugate for DMD (SRP‐5051) has been completed and a phase II is recruiting to determine the optimal dose.

#### Lipids

Conjugation of hydrophobic compounds such as cholesterol to ONs can improve delivery *in vitro* by promoting endosomal release (Wang *et al,* 2019) and results in longer plasma half‐life and accumulation in the liver upon systemic administration (Osborn *et al,* 2019). Such modifications may enhance delivery, mainly to the liver, but also to peripheral tissues such as muscle (Prakash *et al,* 2019), via passive targeting by increasing the binding affinity of ONs to plasma proteins and/or via active targeting by hijacking endogenous lipid transport pathways (Osborn *et al,* 2019).

#### Receptor ligands

Tissue‐specific active targeting may be achieved through conjugation of ONs to receptor ligands that facilitate specific binding to receptors on the target cells and mediate tissue‐specific delivery. A wide variety of receptor ligands have been investigated, including carbohydrates, peptides/proteins, aptamers, antibodies/antibody fragments and small molecules), and several feasible receptor‐ligand systems have been identified.

Perhaps the most successful tissue targeting ligand is trimeric N‐acetyl galactosamine (GalNAc) (Lee *et al,* 1984). GalNAc binds to the asialoglycoprotein receptor (AGPR), which is abundantly expressed in the liver (Schwartz *et al,* 1980). This high affinity‐binding ligand has been directly conjugated to ONs and siRNA and provides highly specific and effective delivery to hepatocytes (Matsuda *et al,* 2015; Janas *et al,* 2018; Debacker *et al,* 2020). Another striking example is the glucagon‐like peptide‐1 (GLP1) receptor (GLPR1) system for specific targeting of pancreatic β cells (Muller *et al,* 2019). Recent studies showed that GLP1‐ON conjugates are specifically taken up by GLPR1‐expressing cells in the pancreas, including isolated pancreatic islets, and induce strong accumulation and activity in pancreatic β cells in a ligand‐dependent manner upon systemic delivery in mice (Ammala *et al,* 2018).

#### Antibodies

A promising recent development in chemical conjugates is antibody–RNA conjugates (ARCs). ARCs typically include monoclonal antibodies, or antibody fragments, with functional ONs, and they have been used for imaging and protein detection. However, antibodies can also be used as a delivery agent for therapeutic ONs. An antibody fragment specific for the transferrin receptor, which is involved in intracellular transport of iron‐laden transferrin, has been used to target siRNA towards skeletal and cardiac muscle tissues (Sugo *et al,* 2016). Companies are taking this technology forward for diseases such as myotonic dystrophy and Duchenne muscular disease.

#### Aptamers

Aptamers have been shown to mediate delivery of therapeutic ONs as aptamer‐ON conjugates, or within nanoparticle formulations (Catuogno *et al,* 2016; Soldevilla *et al,* 2018). The first aptamer‐siRNA chimeras targeted prostate‐specific membrane antigen‐expressing cancer cells to deliver apoptosis‐inducing siRNAs (McNamara *et al,* 2006). Further development of aptamer‐ONs involved chemical modifications to protect the ONs from nuclease degradation and increase their plasma half‐life. Aptamer‐ONs have since shown effective *in vivo* delivery of miRNAs, antagomirs, ASOs and bi‐modular miRNA‐antagomirs within preclinical cancer models (Catuogno *et al,* 2015; Esposito *et al,* 2016; Soldevilla *et al,* 2018).

### Carrier‐based delivery systems

The pharmacological properties of carrier‐based delivery systems are largely independent of the physicochemical properties of the ON cargo, and instead depend on the properties of the delivery system. Therefore, the desired properties can be built into them via formulation design, resulting in multifunctional advanced drug delivery systems. These delivery systems may serve (often simultaneously) many different purposes, including (i) protecting the ON cargo from premature degradation, (ii) increasing the effect duration and (iii) enhancing the targeting. This improved targeting can either occur via passive or active targeting. Passive targeting exploits the microanatomical features of tissues, for instance, tissues with enhanced permeability and retention, or tissues with discontinuous/fenestrated epithelium. For active targeting, delivery systems are decorated with active targeting ligands. Particulate carrier‐based delivery systems also facilitate intracellular delivery by enhancing cellular uptake, intracellular trafficking and endosomal escape. In that way, the dose reaching non‐target tissues and/or the toxicological targets may be reduced, whereas the dose reaching the pharmacological target may be increased. The net result is an improved drug therapeutic index. The complexity of these systems leads to new challenges in the development, for example with respect to cost, manufacturability, safety, quality assurance and quality control.

Reflecting the immense interest in delivery of therapeutic ONs, a plethora of nanocarrier types have been investigated for delivery purposes, such as gold nanoparticles (Ding *et al,* 2014; Morgan *et al,* 2019), mesoporous silica (Steinbacher & Landry, 2014; Cha *et al,* 2017) and other inorganic nanocarriers (Malmsten, 2013). Yet, the current focus seems to be on lipid‐, polymer‐ and peptide‐based delivery systems and hybrids of these, which are described further below (Fig 4).

#### Lipid‐based delivery systems

The recent approval of patisiran (Table 3) (Suhr *et al,* 2015), together with improvements in manufacturability brought about by the introduction of microfluidics, has reinforced interest in lipid‐based delivery systems to the scientific community and pharmaceutical industry. The term lipid nanoparticles (LNPs) is used generically below to describe ON‐loaded lipid‐based delivery systems, because the structural complexity of most lipid‐based nanocarriers complicates their further classification into, for example, liposomes and solid lipid nanoparticles.

Cationic lipids entrap ONs via attractive electrostatic interactions (Felgner *et al,* 1987), and highly efficient commercial *in vitro* transfection reagents are based on cationic lipids. However, as systemic toxicity of cationic lipids is often dose‐limiting for *in vivo* application, ionisable lipids that are positively charged at low pH, *e.g*. during LNP manufacture, and typically neutral at physiological pH, are favoured (Semple *et al,* 2001). Today, a vast number of ionisable lipids have been developed covering a wide range of different structures. These include, among others, lipidoids (Akinc *et al,* 2008; Dong *et al,* 2014) and the ionisable lipid DLin‐MC3‐DMA (Jayaraman *et al,* 2012), which is considered the gold standard of ionisable cationic lipids. In general, they display headgroups containing tertiary amines, which are protonated under acidic conditions and uncharged at neutral pH. The hydrophobic lipid tails stabilise the LNP structure during formation and in formulation via hydrophobic interactions.

Clinically approved patisiran contains DLin‐MC3‐DMA, helper lipids (Kulkarni *et al,* 2019) and PEG‐lipid encapsulating siRNA directed against transthyretin (TTR) mRNA (Adams *et al,* 2018). The PEG lipid stabilises the LNPs during manufacturing and storage, and it increases the circulation half‐life. However, PEG lipids inhibit cellular transfection; hence, they are designed to rapidly diffuse from the LNPs after IV administration (Chen *et al,* 2016). The LNPs are passively targeted to the liver (Shi *et al,* 2011), and the size of the LNPs permits delivery through the fenestrated endothelium in the liver to the underlying hepatocytes (Chen *et al,* 2016). In addition, active hepatocyte targeting has been shown to occur via surface adsorption of apolipoprotein E, which targets the LNPs to the internalising low‐density lipoprotein receptor expressed on hepatocytes (Akinc *et al,* 2010; Chen *et al,* 2016). After cellular uptake, endosomal escape of siRNA into the cytosol may be facilitated via interactions between the re‐protonated ionisable cationic lipid in the acidic endosomal environment and anionic endogenous lipids in the endosomal membrane (Habrant *et al,* 2016).

Exosomes are particular lipid‐based nanocarriers (Barile & Vassalli, 2017). These nanosized vesicles are shed from the cells, encapsulating part of the cellular cytoplasm in the process (Pathan *et al,* 2019). They are remarkable in their biocompatibility and potential for highly specific active targeting through surface display of endogenous cellular ligands. The main challenges for using exosomes as delivery systems are (i) reproducible, large‐scale production and (ii) effective loading of drugs. Additionally, exosome heterogeneity is caused by their natural content of proteins and nucleic acids derived from the host cell (Willms *et al,* 2018; Jeppesen *et al,* 2019). This complicates their use as therapeutic delivery agents. The therapeutic promise of exosomes has been extensively reviewed elsewhere (Wiklander *et al,* 2019).

#### Polymer‐based delivery systems

Although less clinically advanced, polymer‐based systems are also interesting carriers for ON delivery, largely due to the chemical flexibility of polymers, in particular synthetic polymers (Fig 4) (Freitag & Wagner, 2020). Both monomer sequence and side/end group functionalities can be engineered. Additionally, polymeric nanocarriers exhibit high structural integrity and stability during storage.

One polymer with high biocompatibility that has been studied and used extensively is the copolymer poly(lactic‐co‐glycolic acid) (PLGA) (Rezvantalab *et al,* 2018). For small‐molecule drugs, highly efficient encapsulation in polymeric nanoparticles can be achieved, *e.g*. through miniemulsion‐based synthesis, followed by *in situ* polymerisation (Fusser *et al,* 2019). However, due to their negative charge, anionic ONs cannot be encapsulated using this approach. Instead, encapsulation can be achieved through attractive electrostatic interactions between the anionic ONs and polycationic polymers. Dendrimers are hyperbranched polymers, which are well suited for this purpose because they can complex many ON molecules. Several cationic polymers have been used, including poly(amidoamine), poly(propyleneimine) and poly(L‐lysine) [reviewed by (Mignani *et al,* 2019)].

Among the synthetic polymers, polyphosphazenes are notable in their high biocompatibility and chemical flexibility, and they have successfully been used to deliver therapeutic ONs (Peng *et al,* 2016; Hsu *et al,* 2020). Polyphosphazenes can be tailored to exhibit responsivity to external (bio)chemical stimuli (Teasdale, 2019), *e.g*. local pH. This allows for a targeted release of the cargo at the desired site of action. Complementing the use of synthetic polymers, there is long‐standing interest in the use of naturally occurring biopolymers for ON encapsulation; the most notable example is the use of the polycation chitosan, often in complex with another, anionic polymer, *e.g*. PLGA (Taetz *et al,* 2009) or alginate (Lee & Mooney, 2012).

Recently, there has been significant interest in lipid–polymer hybrid nanoparticles (Thanki *et al,* 2017). These hybrids combine desirable properties from both nanoparticle types, i.e. the serum stability of PLGA‐based matrix system with the biocompatibility and high loading capacity of ONs in delivery systems based on cationic lipids.

#### Peptide‐based delivery systems

CPPs represent another group of compounds that have been also successfully used as a carrier‐based drug delivery system (Lehto *et al,* 2016). In this context, formation of CPP/ON nanoparticles is driven by electrostatic and hydrophobic interactions between cationic CPPs and anionic ONs. Compared with directly conjugated CPP‐ONs, peptide‐based vectors are more amphipathic and usually carry additional chemical modifications that make them compatible with encapsulating ONs. Commonly, such modifications include incorporation of various hydrophobic modifications, such as fatty acid derivatives, to the CPP sequences, which increase the stability of the formulation and enhance their cellular uptake and endosomal escape. Various types of CPPs have demonstrated considerable potential for ON delivery in a nanoparticle‐based format, including MPG and PepFect peptide derivatives [reviewed in (Boisguerin *et al,* 2015; Lehto *et al,* 2016)].

#### Antibody complexation delivery systems

Antibodies are another promising form of carrier delivery system used both as direct conjugates or unconjugated carriers. As unconjugated carriers, antibodies or antibody fragments have been fused with either avidin or protamine peptide. Taking advantage of the natural avidin–biotin complexation system, antibody–avidin fusion molecules bind to biotinylated ONs (Penichet *et al,* 1999). The peptide protamine is a positively charged RNA‐binding peptide, which binds to siRNA and condenses it into antibody–siRNA complex (Song *et al,* 2005). This system has been used to link cytotoxic siRNAs with Her2‐positive cancer cell‐targeted antibodies (Yao *et al,* 2012). Like all complexation systems, these two systems have the advantage of an established target‐specific antibody carrier, which can easily be complexed with any siRNA.

## Model systems for oligonucleotide development

Successful development of ON‐based drugs depends on detailed knowledge about pharmacokinetic (PK) and pharmacodynamic (PD) properties. PK/PD analyses describe the relationship between PK (drug concentration in the organism) and PD (the organism's biological response to the drugs) in a time‐dependent manner (Negus & Banks, 2018). PK/PD modelling and simulations are used to rapidly characterise the efficacy and safety of drugs, and PK/PD simulation models containing *in vitro* and *in vivo* preclinical studies can anticipate potential risks in humans (Li *et al,* 2016). The use of predictive model systems for PK/PD analyses save time, costs and minimise the need for *in vivo* studies, facilitating the translation from bench to bedside.

### Methodologies for in vitro testing of oligonucleotides

*In vitro* models can be implemented to test pharmacological activity, transfection efficiency, hepatotoxicity and intracellular half‐life. However, it is usually difficult to correlate *in vitro* findings to preclinical and clinical *in vivo* findings (Table 2). Novel technologies, such as reprogramming patient‐derived cells into induced pluripotent stem cells (iPSCs) (Takahashi & Yamanaka, 2006; Takahashi *et al,* 2007) and genome editing techniques to make isogenic cell lines, have revolutionised the field (Ran *et al,* 2013). Two‐dimensional (2D) and three‐dimensional (3D) cell cultures, including organoids, are used to improve the understanding of pathological disease mechanisms, as well as ON efficacy studies. One example of successful translation from a 3D‐model to a clinical trial is sepofarsen for the treatment of the inherited retinal disease Leber congenital amaurosis (LCA) (Collin *et al,* 2012; den Hollander *et al,* 2006). Combining patient‐derived retinal organoids with toxicity studies in non‐human primates (NHPs) was sufficient to initiate a phase I/II clinical trial (NCT03140969, NCT03913143) (Cideciyan *et al,* 2019). The eye is an exceptional target organ, given its isolated and immune‐privileged status, which allows for translation of results from organoids in culture to the human eye. However, for other (multi‐)organ diseases, establishing predictive cellular models to mimic the functions of entire organs remains a challenge.

**Table 2:** Comparison of different disease models.

	*In vivo*	*In vitro*	3D Organoids	Organs‐on‐chips
Human‐derived tissue	No	Yes	Yes	Yes
Personalised medicine	No	Yes	Yes	Yes
Realistic microenvironment	Yes	No	Yes	Yes
Organ‐level function	Yes	Limited	Potentially/Limited	Potentially
Real‐time readouts	No	Limited	Limited	Yes
High‐throughput testing	No	Yes	Limited	Possibly
Pharmacodynamics / ‐kinetics	Yes	No	No/Limited	Potentially

An interesting alternative to 2D and 3D tissue culture techniques is the microfluidics‐based *organ‐on‐chip* technology (van der Meer & van den Berg, 2012), which consists of micro‐engineered iPSC‐derived models that combine the advantages of current *in vitro* and *in vivo* models. The technology breaks down organs into the most essential components, including biological barriers, for drug delivery, efficacy, toxicity and PK/PD studies. Organ‐on‐chips reproduce the interaction between cultures of multiple tissue types using microfluidic channels and chambers (Huh *et al,* 2010; Kim *et al,* 2012; Westein *et al,* 2013). This interaction can be monitored in real time to study the PK/PD of a specific drug as well as drug–drug interactions (Lee *et al,* 2017; Shinha *et al,* 2020). For instance, the PK/PD evaluation of terfenadine (a type of antihistamine) has been assessed by using a cellular model combining heart and liver cells in two interconnected chambers. This model, combined with microelectrode arrays, also contributed to predict the potential cardiotoxicity of the drug (McAleer *et al,* 2019). Interestingly, recent drug permeability studies in blood–brain barrier (BBB)‐on‐chip models were found to be more predictive compared with existing *in vitro* models (van der Helm *et al,* 2016). Other cellular models under development include retina‐on‐chip (Achberger *et al,* 2019; Seo *et al,* 2019) and lung‐on‐chip (Huh *et al,* 2010) models. Mimicking the function of entire organ(s) in a dish by combining several cell types in a single device may have valuable potential for drug screening and development, as well as PK/PD and toxicity studies. In the future, organ‐on‐chip models might, to some extent, replace experimental animal models.

### Investigation of PK/PD properties in vivo

*In vivo* models have been extensively used for dose‐finding studies. PK properties are largely comparable across multiple species including mouse, rat, NHP and human (Yu *et al,* 2009; Geary *et al,* 2015). Hence, cross‐species PK/PD relationships are very valuable for the prediction of human dosing. Animal models have been vital for determining *in vivo* efficacy of ONs, tissue‐specific delivery, and optimising the route of administration for systemic and neurological diseases (Schoch & Miller, 2017; Buijsen *et al,* 2019). Preclinical *in vivo* testing in a transgenic mouse model for SMA predicted the enhanced benefit of treating pre‐symptomatic stages of the disease, which was later validated in the clinic.

However, detailed knowledge of the disease model is vital for interpreting data: A study in the *mdx* mouse model for DMD of the PK/PD of 2ʹ‐*O*Me ONs for DMD revealed higher ON levels in dystrophin‐deficient muscle fibres than in healthy fibres, as well as an enhanced exon skipping efficiency (Heemskerk *et al,* 2010). However, ON efficiency was lower in clinical trials in DMD patients, potentially due to a better regenerative capacity in mice. Also, animal models may not always reciprocate the human condition due to the different genomic context of the mutations, even when using humanised animal models. This is evident for pre‐mRNA splicing, which is differentially regulated between tissues, organs and species (Rivera‐Barahona *et al,* 2015). Between tissues, DNA variants have been observed to affect pre‐mRNA splicing, complicating the interpretation of *in vitro* studies. An example is the aforementioned deep‐intronic change underlying LCA: while lymphoblastoid and fibroblast cells derived from patients suggested a hypomorphic effect (Garanto *et al,* 2016), reprogrammed patient‐derived iPSCs differentiated towards a retinal fate revealed that the percentage of aberrantly spliced mRNA was highly increased in photoreceptor cells, explaining the retinal phenotype observed in LCA patients (Parfitt *et al,* 2016). Follow‐up studies revealed that a pseudoexon present in humans was differentially recognised in cell lines derived from other species (Garanto *et al,* 2015). Thus, care is warranted when selecting a model system for assessing the effects of a certain genetic variant, as well as for the development of splice‐modulation therapies.

## Safety assessment of oligonucleotide‐based therapeutics

**Figure 5:**
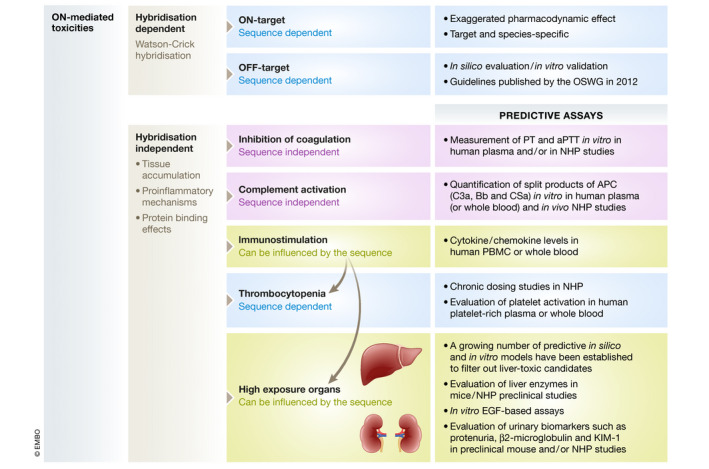
ASO mediated toxicities Schematic representation of the most common ON‐mediated toxicities, which are mainly classified as hybridisation‐dependent (Watson–Crick hybridisation) or hybridisation‐independent effects (tissue accumulation, proinflammatory mechanisms and/ or protein binding effects). Some of them are strictly class specific (sequence independent), while others can be influenced by the sequence (sequence specific).

While new chemistries and delivery technologies might lead to higher efficacy, it is important to screen for potential side effects in early phases of preclinical development to avoid subsequent failure. Toxicological aspects of therapeutic ONs have been comprehensively summarised previously (Andersson & den Besten, 2019). The Oligonucleotide Safety Working Group (OSWG) has also published extensive guidelines for assessing the various aspects of ON safety. Our understanding of ON‐mediated toxicity increases as more preclinical and clinical data become available. While the concept of class toxicity appears nuanced in the light of the expanding knowledge on various chemistries, ON‐related side effects still falls under two main categories: (i) hybridisation‐dependent effects, including on‐ and off‐target effects, and (ii) hybridisation‐independent effects, mostly caused by protein‐binding properties (Fig 5).

### Hybridisation‐dependent effects

On‐target safety, also referred to as exaggerated pharmacology, relates to the possible toxicities induced by excessive or prolonged activity of the ON in target or non‐target organs. These effects are considered rare and are generally discovered in preclinical studies. However, due to the sequence‐specific action of ON‐based drugs, target sequences may not be conserved across species. Therefore, human sequences might not display efficacy in rodents or NHPs; hence, species‐specific surrogates are needed for on‐target risk assessment (Levin & Henry, 2008).

Off‐target effects correspond to the potential toxicities associated with ON hybridisation to unintended RNA targets (complete or partial complementarity). They have increased with the development of high‐affinity chemistries, *e.g*. LNA, tcDNA and constrained ethyl (cEt), which allow the use of much shorter sequences. Off‐target effects are of particular concern for gapmer ONs and siRNA, which aim at downregulating their targets, as they could downregulate the expression of unintended ones (Fedorov *et al,* 2006; Burel *et al,* 2016). Several studies have characterised off‐target effect‐associated mechanisms and described elegant ways to reduce risks and improve the design of specific gapmers and siRNAs (Hagedorn *et al,* 2017; Janas *et al,* 2018). In contrast, splice‐switching ASOs must bind specific splicing regulatory elements to be efficacious, and they are therefore less likely to induce off‐target effects. With the development of more stable ONs and efficient delivery systems, systemic administrations might distribute to target but also non‐target tissues; hence, off‐target effects should be carefully evaluated during preclinical development. The guidelines published by the OSWG for assessing off‐target effects recommend (i) *in silico* evaluation, (ii) interpretation of *in silico* hits using auxiliary data (*e.g*. time‐ and spatiotemporal‐dependent expression of off‐target RNA) and (iii) *in vivo* evaluation of ON drugs (Lindow *et al,* 2012).

### Hybridisation‐independent effects

Most ON‐mediated toxicities are not caused by Watson–Crick base pairing to RNA, but are rather a result of ON–protein interactions and therefore depend on the chemistry and/or the delivery system. Single‐stranded PS‐modified ON display particularly high protein binding affinities, and the majority of the hybridisation‐independent effects have thus been reported for this class of ON, as opposed to siRNA containing less PS‐modified residues.

#### Inhibition of blood coagulation

Inhibition of the intrinsic blood coagulation pathway is a well‐documented side effect of the PS chemistry (Henry *et al,* 1997b; Echevarria *et al,* 2019). It is considered a class effect, modulated by interactions of the ON with plasma proteins in a sequence‐independent way. The PS modification selectively prolongs the partial thromboplastin time at low plasma concentrations by inhibiting the tenase complex. However, at high plasma concentrations, both the intrinsic and extrinsic pathways are affected, suggesting additional inhibitory effects (Sheehan & Lan, 1998). Prolongation of clotting times is correlated with the maximal plasma concentration (*C*_max_) of circulating ONs, and it has not been associated to relevant clinical signs, as it can be controlled by dose reduction or by extending infusion times. Nevertheless, it should be included in screening studies, which can be performed both *in vivo* and *in vitro* in mouse, NHP and human serum, respectively, since results can be extrapolated across species (Andersson & den Besten, 2019).

#### Complement activation

Systemic administration of PS‐modified ONs has been reported to activate the alternative complement pathway as a consequence of plasma protein binding (Henry *et al,* 2002). Although this hybridisation‐independent effect is mainly related to the ON chemistry (class effect), unexpected complement activation has been observed with some sequence specificity, as in the case of tcDNA (Aupy *et al,* 2020). Activation of the alternative complement pathway has been thoroughly studied in NHP models, which are particularly sensitive (Henry *et al,* 2016). The effect is dependent on the plasma concentration and can be controlled by increasing the IV infusion time to reduce the C_max_. PS‐modified ONs have been shown to interact directly with plasma factor H, which is a negative regulator of the complement cascade that reduces the free levels of inhibitor, permitting uncontrolled amplification of the cascade and release of split products such as Bb and anaphylotoxins C3a and C5a (Henry *et al,* 1997a). Complement can be activated similarly at every dose; hence, chronic administration of toxic ONs can result in C3 depletion, eventually leading to altered complement function, secondary inflammation and vasculitis (Engelhardt *et al,* 2015; Shen *et al,* 2016; Andersson & den Besten, 2019). Although humans appear less sensitive to complement activation, it is recommended to routinely evaluate complement activation in preclinical safety studies of new ON‐drug candidates in NHPs.

Complement activation can be assessed *in vitro* in NHP or human serum, or whole blood, to measure split products of the alternative complement pathway (Bb, C3a and C5a). Nevertheless, one should be cautious when interpreting the results, as it is difficult to extrapolate and predict dose–response relationships (Andersson & den Besten, 2019).

#### Immunostimulation

ON‐induced immunostimulation is a complex side effect that depends on several aspects, including chemistry and nucleotide sequence (Krieg, 1998; Agrawal & Kandimalla, 2004). ONs can activate the innate immune system through binding to pattern‐recognition receptors (PRRs) such as the Toll‐like receptors (TLRs). Activation of the innate immune system by CpG‐containing ONs is comparable to that observed for bacterial DNA and CpG‐containing ONs are used for cancer and autoimmune disease therapies as well as vaccine adjuvants (Krieg & Davis, 2001; Krieg, 2006; Kline & Krieg, 2008). However, the immunostimulatory activity of ONs designed for antisense purposes constitutes a potential side effect. In this regard, modified ONs with 2ʹ‐ribose modifications, 5‐methyl cytosine residues, or without CpG motifs, have been designed to avoid TLR9 activation. Additional studies have demonstrated that CpG‐free, PS‐modified ONs can also elicit proinflammatory responses, although the molecular mechanism is still debated (Vollmer *et al,* 2004; Senn *et al,* 2005; Younis *et al,* 2006). Of note, immunostimulatory effects have never been reported for ONs with neutral backbones, *e.g*. PMOs (Zhang *et al,* 2015). Rodents are particularly sensitive to immune stimulation. Mice treated with high doses of PS‐ONs display increased levels of circulating cytokines (IL‐1b, IL‐6, interferon, tumour necrosis factor‐α) and chemokines, as well as proliferation of B‐lymphocytes (Monteith *et al,* 1997). Although generally less critical, some significant inflammatory responses, *e.g*. vasculitis, related to complement activation mediated by PS‐ONs, have been described in NHP studies (Levin & Henry, 2008; Engelhardt *et al,* 2015; Frazier, 2015; EMA, 2016). Differences in immune response between species have been attributed to the differential sequence, expression and function of the germline‐encoded PRRs (Barchet *et al,* 2008).

In clinical trials, inflammatory adverse effects may manifest as flu‐like symptoms and injection site reactions following subcutaneous (SC) administration (Rudin *et al,* 2001; Thomas *et al,* 2013; Voit *et al,* 2014). Understanding the underlying mechanisms of therapeutic ON‐mediated induction of proinflammatory adverse effects has facilitated the design of safer and more potent sequences that are efficacious at lower doses. Nevertheless, some sequences still display unexpected toxicity, and specific screening for immunostimulatory adverse effects is recommended. In addition to *in vivo* studies in rodents and NHPs, proinflammatory evaluation is usually performed *in vitro* using human peripheral blood mononuclear cells or whole blood (Apter *et al,* 1990; Lankveld *et al,* 2010).

The potential immunogenicity of ONs is poorly documented but recent data show that anti‐drug antibodies (ADAs) are present in NHPs and humans (Andersson & den Besten, 2019). More than 30 and 70% of patients treated with drisapersen and mipomersen, respectively, were found positive for ADA after 24 weeks of treatment. Recently, ADA plasma levels were shown to increase both in monkeys and humans, while no impact on efficacy and safety was reported (Bosgra *et al,* 2019; Yu *et al,* 2020).

Formulations based on nanoparticles administered IV may also induce infusion‐related reactions (IRRs), *e.g*. hypersensitivity, evident as flu‐like symptoms and even cardiac anaphylaxis (Szebeni, 2018). Hence, before IV infusion of patisiran, patients are required to be premedicated with IV antihistamines (H1/H2 blockers), IV corticosteroid and oral acetaminophen or paracetamol to suppress IRRs. Mild to moderate IRRs were observed in a phase III trial of patisiran in approximately 20% of the patients, who were all premedicated, the incidence of which decreased over time (Adams *et al,* 2018). In contrast, premedication is not required before administration of ONs and GalNAc–siRNA conjugates.

#### Thrombocytopenia

ON‐associated thrombocytopenia is an occasional event that has been observed in rodents and NHPs, as well as in three recent clinical trials with unrelated PS‐ONs [volanesorsen (FDA, 2018), inotersen (Benson *et al,* 2018; Mathew & Wang, 2019) and drisapersen (EMA, 2016; Goemans *et al,* 2016)]. The exact underlying mechanisms of thrombocytopenia is still debated, and several immune and non‐immune mediated mechanisms have been proposed. Direct activation of platelets by PS‐ONs through the binding to platelet receptors has been demonstrated (Flierl *et al,* 2015; Sewing *et al,* 2017). In addition, a heparin‐induced thrombocytopenia‐like mechanism through the induction of anti‐platelet factor 4 IgG antibodies has also been proposed, based on the binding of nucleic acids to platelet factor 4 (Jaax *et al,* 2013), although contradictory results have been reported. A recent study suggests that sequestration of platelets in the liver and spleen occurs through the activation of monocytes, but not platelets, and is accompanied by increased serum IgM levels (Narayanan *et al,* 2018). In most cases, thrombocytopenia after treatment with ONs is mild to moderate and reversible. The number of platelets does not drop below the normal limit during treatment and normalises after withdrawal from treatment. However, a concerning and severe decline (< 50,000 platelets/µl) has been observed in NHP studies after repeated dosing (Henry *et al,* 2017). To date, severe thrombocytopenia has not been reported for siRNA drugs, neither in preclinical studies nor in clinical trials, but encapsulation of siRNA to LNPs has been shown to cause thrombocytopenia in rats, presumably induced by the cationic lipid molecules themselves (Chi *et al,* 2017).

### High‐exposure organs

Following IV administration and independently of the chemistry, the highest concentrations of ONs are found in the liver and the kidneys, which are considered high‐exposure organs (Fig 5). The toxicities observed in these organs are not necessarily associated with the accumulation of ONs *per se* but can also be due to sequence‐specific effects. Accumulated ONs are often apparent as basophilic granules (ONs in lysosomal compartments) in tissue sections. However, these effects are regarded as non‐adverse because of their reversible nature upon termination of treatment. In contrast, acute toxicities characterised by large areas of necrosis, pronounced elevation of liver enzyme levels, morbidity and mortality have been reported for some high‐affinity gapmers after a single or few doses in mice (Hagedorn *et al,* 2013; Burdick *et al,* 2014). The mechanisms underlying these sequence‐specific acute toxicities may be accumulation of RNase H‐cleaved mRNA products and/or protein interactions (Burel *et al,* 2016; Sewing *et al,* 2016; Shen *et al,* 2019). While the screening for these acute toxicities previously relied on *in vivo* studies assessing levels of liver enzymes following IV administration in rodents, a growing number of predictive *in silico* (Hagedorn *et al,* 2013; Burdick *et al,* 2014) and *in vitro* models (Sewing *et al,* 2016; Dieckmann *et al,* 2018) have been established.

Renal lesions are generally restricted to the proximal tubules and appear only in animals treated with much higher ON doses than the clinically relevant doses. No clinically significant renal dysfunction was reported in a large retrospective study of 2ʹ‐MOE gapmer trials (Crooke *et al,* 2018). Renal toxicity was mostly regarded as accumulation‐related toxicity and primarily sequence unspecific until more acute tubular lesions were reported with high‐affinity ONs, *e.g*. LNAs (Engelhardt *et al,* 2015). Beyond the classical biomarkers for renal injury, *e.g*. increased excretion of β2‐microglobulin and kidney injury molecule‐1, a predictive epidermal growth factor‐based assay has recently been developed to exclude this type of nephrotoxic candidates (Moisan *et al,* 2017).

## Approved oligonucleotide‐based therapeutics

Advances in therapeutic ON technology in recent decades provide a unique opportunity for addressing previously inaccessible drug targets (Bennett *et al,* 2017). Since the approval of fomivirsen in 1998 by the FDA for treating cytomegalovirus (CMV) retinitis (Marwick, 1998), 11 ON‐based drugs have received marketing authorisation to be used in humans, and two additional ON drugs have received positive opinion for marketing by the EMA (Table 3). Here, we discuss approved ON therapeutics according to their functional modalities.

Fomivirsen (Vitravene) is a 21‐mer PS DNA‐based ON developed for treating CMV retinitis patients, especially those with acquired immunodeficiency syndrome (AIDS) (Vitravene Study G, 2002). This first‐generation ASO targets the human CMV major immediate‐early gene mRNA for RNAse H degradation (Geary *et al,* 2002). Fomivirsen is delivered locally by IVT administration and hence does not require a delivery agent. While a second‐generation 2ʹ‐MOE‐based gapmer sequence (ISS 13312) was in clinical development (Henry *et al,* 2001), Novartis discontinued development and withdrew marketing (Wathion, 2002). The number of CMV retinitis cases had decreased dramatically due to the development of highly active antiretroviral therapy. Nevertheless, fomivirsen was a success and established ON therapies as viable for clinical development.

**Table 3:** Clinically approved oligonucleotides.

Approval date		Drug name	Disease	Target	ASO sequence 5′–3′	Administration route/target tissues
FDA	EMA					
**RNaseH**						
26 August 1998	29 July 1999	Fomivirsen (Vitravene)	Cytomegalovirus retinitis in immunocompromised patients	CMV major immediate‐early gene mRNA	dGs‐dCs‐dGs‐dTs‐dTs‐dTs‐dGs‐dCs‐dTs‐dCs‐dTs‐dTs‐dCs‐dTs‐dTs‐dCs‐dTs‐dTs‐dGs‐dCs‐dG	IVT / eye
29 January 2013	refused authorisation	Mipomersen (Kynamro)	Homozygous familial hypercholesterolemia	Apolipoprotein B‐100	Gs*‐mCs*‐mCs*‐Ts*‐mCs*‐dAs‐dGs‐dTs‐dmCs‐dTs‐dGs‐dmCs‐dTs‐dTs‐dmCs‐Gs*‐mCs*‐As*‐mCs*‐mC*	SC / liver
05 October 2018	05 July 2018	Inotersen (Tegsedi)	Hereditary transthyretin amyloidosis	Transthyretin	mTs*‐mCs*‐mTs*‐mTs*‐Gs*‐dGs‐dTs‐dTs‐dAs‐dmCs‐dAs‐dTs‐dGs‐dAs‐dAs‐dAs‐mTs*‐mCs*‐mCs*‐mC*‐3′	SC / liver
Under review	03 May 2019	Volanesorsen (Waylivra)	Familial chylomicronemia syndrome, hypertriglyceridemia and familial partial lipodystrophy	Apolipoprotein CIII	As*‐Gs*‐mCs*‐Ts*‐Ts*‐dmCs‐dTs‐dTs‐dGs‐dTs‐dmCs‐dmCs‐dAs‐dGs‐dmCs‐Ts*‐Ts*‐Ts*‐As*‐T*	SC / liver
**Splice modulation**						
01 June 2019‡	N/A	Milasen	Neuronal ceroid lipofuscinosis 7, Batten's disease	MFSD8 exon 6	As*‐As*‐Ts*‐Gs*‐Ts*‐Ts*‐As*‐Gs*‐Ts*‐Gs*‐mCs*‐Ts*‐Ts*‐Gs*‐Ts*‐Ts*‐Gs*‐As*‐Gs*‐Gs*‐Gs*‐mC*	IT / CNS
19 September 2016	refused authorisation	Eteplirsen (Exondys51)	Duchenne muscular dystrophy	Dystrophin exon 51	*CTCCAACATCAAGGAAGATGGCATTTCTAG*	IV / skeletal muscle
12 December 2019	Under review	Golodirsen (Vyondys 53)	Duchenne muscular dystrophy	Dystrophin exon 53	*GTTGCCTCCGGTTCTGAAGGTGTTC*	IV / skeletal muscle
August 2020	Under review	Viltolarsen† (Viltepso)	Duchenne muscular dystrophy	Dystrophin exon 53	*CCTCCGGTTCTGAAGGTGTTC*	IV / skeletal muscle
23 December 2016	30 May 2017	Nusinersen (Spinraza)	Spinal muscular dystrophy	Survival motor neuron 2 exon 7	mTs*‐mCs*‐As*‐mCs*‐mTs*‐mTs*‐mTs*‐mCs*‐As*‐mTs*‐As*‐As*‐mTs*‐Gs*‐mCs*‐mTs*‐Gs*‐G*	IT / CNS
**Aptamer**						
17 December 2004	31 January 2006	Pegaptanib (Macugen)	Age‐related macular degeneration	Vascular endothelial growth factor	40 kDa PEG‐5′‐C^F^‐G^‐G^‐A‐A‐U^F^‐C^F^‐A^‐G^‐U^F^‐G^‐A^‐A^‐U^F^‐G^‐C^F^‐U^F^‐U^F^‐A^‐U^F^‐A^‐C^F^‐A^‐U^F^‐C^F^‐C^F^‐G^‐3′‐3′‐dT‐5′	IVT / eye
**RNAi**						
10 August 2018	27 August 2018	Patisiran (Onpattro)	Hereditary transthyretin amyloidosis	Transthyretin	5′‐G‐U^‐A‐A‐C^‐C^‐A‐A‐G‐A‐G‐U^‐A‐U^‐U^‐C^‐C^‐A‐U^‐dT‐dT‐3′ 3′‐dT‐dT‐C‐A‐U^‐U‐G‐G‐U‐U‐C‐U‐C‐A‐U^‐A‐A‐G‐G‐U‐A‐5′ LNP formulated	IV / liver
20 November 2019	02 March 2020	Givosiran (Givlaari)	Acute hepatic porphyria	Aminolevulinate synthase 1	5′‐Cs^‐As^‐G^‐A^‐A^‐A^‐G^F^‐A^‐G^F^‐U^‐G^F^‐U^‐C^F^‐U^‐C^F^‐A^‐U^‐C^‐U^‐U^‐A^‐3′ 3′‐Us^‐Gs^‐G^‐U^F^‐C^‐U^F^‐U^‐U^F^‐C^‐U^F^‐C^‐A^F^‐C^‐A^F^‐G^‐A^F^‐G^‐U^F^‐A^‐G^F^‐As^F^‐As^F^‐U^‐5′ GalNAc Conjugate	SC / liver
	16 October 2020¥	Lumasiran (Oxlumo)	Primary hyperoxaluria type 1	Hydroxiacid oxidase 1	5′‐As^‐Cs^‐C^‐U^‐G^‐A^‐A^‐A^F^‐G^‐U^F^‐A^‐G^‐G^‐A^‐C^F^‐C^F^‐U^‐U^F^‐U^‐A^‐Us^‐As^F^‐U^‐3′ 3′‐Gs^‐As^‐Cs^‐U^‐U^‐U^‐C^F^‐A^‐U^F^‐C^F^‐C^F^‐U^‐G^‐G^‐A^‐A^‐A^‐U^‐A^‐U^‐A^‐5′ GalNAc Conjugate	SC / liver
	16 October 2020¥	Inclisiran	Atherosclerotic cardiovascular disease	Proprotein convertase subtilisin‐kexin type 9	5′‐As^‐C^F^s‐As^‐A^F^‐A^F^‐A^F^‐G^‐C^F^‐A^‐A^F^‐A^‐A^‐C^‐A^F^‐G^‐G^F^‐U^‐C^F^‐U^‐A^‐Gs^‐As^‐A^‐3′ 3′‐U^‐G^‐U^‐U^‐U^‐U^‐C^‐G^‐U^‐U^‐dT‐U^‐G^F^‐U^‐C^F^‐C^‐A^‐G^‐As^‐Us^‐C^‐5′ GalNAc Conjugate	SC / liver

s, phosphorothioate linkage; *, 2′‐MOE; d, 2′‐deoxy; m, 5‐methyl; ^F^, 2′‐F; ^, 2′‐OMe; italicised, PMO; † Viltolarsen approval by Japanese Ministry of Health Labour and Welfare, 25 March 2020; ‡ Milasen approved by FDA for clinical testing only, ¥, lumasiran and inclisiran received positive opinion for marketing by the CHMP.

RNase‐dependent second‐generation ASOs targeting the liver have been approved for polyneuropathy of hereditary transthyretin‐mediated amyloidosis (hATTR) (inotersen) as well as familial chylomicronemia syndrome (FCS), hypertriglyceridemia and familial partial lipodystrophy (volanesorsen), and familial hypercholesterolemia (mipomersen). The rare disease hATTR is linked to missense mutations in the *TTR* gene, which result in TTR protein misfolding. The TTR protein is secreted into the blood and cerebral spinal fluid, and accumulation of amyloid deposits (both wild type and mutant) in tissues causes polyneuropathy, multiorgan dysfunction and cardiomyopathy. Inotersen targets the hepatic expression of both wild‐type and mutant TTR mRNA. Patients treated with inotersen display a reduction in serum TTR protein levels and enhanced quality of life (Benson *et al,* 2018). Volanesorsen, although still awaiting FDA approval at the time of writing this review, was awarded EMA approval in May 2019. By targeting the 3ʹ UTR of apolipoprotein C3 mRNA, volanesorsen reduces the levels of triglycerides and apolipoprotein C3, which represent two known risk factors for cardiovascular disease, while increasing the levels of low‐density and high‐density lipoprotein cholesterol and apolipoprotein B in patients with FCS and hypertriglyceridemia (Graham *et al,* 2013; EMA, 2019). Mipomersen also targets apolipoprotein B‐100 to reduce circulating low‐density lipoprotein cholesterol, which constitutes another major risk factor for cardiovascular disease (Wong & Goldberg, 2014). In contrast to inotersen and volanesorsen, mipomersen was given FDA approval, but EMA authorisation was denied due to safety concerns related to liver toxicity and severe cardiovascular events (EMA, 2012). It has since been discontinued by the FDA and is only available through a restricted risk evaluation and mitigation strategy. All three ON therapies are dosed by SC administration without a delivery agent due to the natural uptake of ONs by the liver. In 2004, the aptamer pegaptanib (Macugen) was approved by the FDA for the prevention of the eye‐related disorder age‐related macular degeneration (Ng *et al,* 2006). Pegaptanib is a covalent conjugate of a highly modified single‐stranded aptamer and two 20‐kDa PEG units. It binds with high specificity and affinity to the extracellular VEGF isoform 165 and blocks its neo‐angiogenic activity (Ruckman *et al,* 1998). Patients dosed with pegaptanib demonstrated reduced vision loss compared with placebo controls (Gragoudas *et al,* 2004). Common to degenerative diseases, early treatment results in improved therapeutic outcome (Gonzales CR & Group VISiONCT, 2005).

Several splice modifying ON‐based drugs are approved to treat the paediatric disorders DMD and SMA, focussing on splice modification and targets tissues beyond liver. The first approved drug, *i.e*. eteplirsen (Exondys51), is a PMO‐based splice‐switching ASO that interacts specifically with *DMD* exon 51, and is used in DMD patients with dystrophin deletions amenable to exon 51 skipping (~14% of patients) (Cirak *et al,* 2011). Dystrophin expression is limited mainly to skeletal and cardiac muscles, and eteplirsen is expected to be most efficacious in skeletal muscle. However, conducive with all PMOs, high accumulation in the kidneys and rapid urine excretion is also expected (Heemskerk *et al,* 2009). The approval of eteplirsen by the FDA was accompanied by controversy due to the trial design and difficulties in quantifying increased expression of dystrophin, which leaves doubt on the efficacy of eteplirsen (Aartsma‐Rus & Arechavala‐Gomeza, 2018). As a result, it was not approved by the EMA. Strengthened by improved clinical trial designs, two additional PMO‐based ASOs have recently been approved for DMD patients amenable for dystrophin exon 53 skipping, *i.e.* golodirsen and viltolarsen, by the FDA, and both the FDA and Japanese Ministry of Health Labour and Welfare, respectively (Dhillon, 2020; Heo, 2020).

The only ON‐based therapeutic approved for a neurological disease is nusinersen, used for the treatment of SMA (Aartsma‐Rus, 2017; Finkel *et al,* 2017; Mercuri *et al,* 2018). Nusinersen targets the alternatively spliced exon 7 of *SMN2* pre‐mRNA, increasing exon inclusion and producing a functional SMN protein. It is administered directly to the cerebral spinal fluid surrounding the spinal cord by IT injection (Hache *et al,* 2016). IT administration directs uptake into the CNS, allowing low doses and circumvention of liver metabolism and kidney excretion. Patients, especially young pre‐symptomatic patients, report extended survival and reaching motor milestones over the expected natural history of the disease. Controversy related to nusinersen is not over efficacy but rather the exceedingly high cost, which has delayed approval and prevented marketing in countries with national health services (Starner & Gleason, 2019).

The success of nusinersen has led to the use of ONs as personalised medicines, exemplified in the development of milasen, which targets a mutation specific to a single patient with a form of Batten’s disease (Kim *et al,* 2019). In this case, the insertion of an SVA (SINE‐VNTR‐Alu) retrotransposon altered the splicing of the major facilitator superfamily domain containing 8 (*MFSD8*) exon 6 into a cryptic splice‐acceptor site. Clinicians followed the preclinical studies and trial designs from the nusinersen studies to accelerate the FDA approval of the clinical study: milasen dosing was initiated 14 months after clinical diagnosis and just 4.5 months after identification of a therapeutic ASO. The patient’s rate of deterioration meant that dosing had to be initiated as soon as possible; hence, the patient was dosed in parallel to toxicology studies in animals. Although therapeutic efficacy in a single patient cannot be defined, milasen reduced the frequency and duration of seizures and potentially diminished the neurodegenerative decline.

Two ON therapeutics based on RNAi, *i.e.* patisiran and givosiran, were approved by the FDA in 2018 and 2019, respectively. Patisiran represents an important milestone, because it is the first marketed drug based on siRNA, launched only 20 years after the discovery of the RNAi mechanism (Fire *et al,* 1998). Like inotersen, patisiran inhibits hepatocyte expression of TTR in patients with hATTR (Adams *et al,* 2018). Patisiran consists of siRNA directed against TTR mRNA formulated as LNPs, which are administered systemically by IV infusion. The latest breakthrough is givosiran, which represents the first approved GalNAc‐siRNA conjugate. Givosiran inhibits hepatic synthesis of delta aminolevulinate synthase 1 (ALAS1) in patients with acute hepatic porphyria (AHP), which is a rare inherited disease of haem biosynthesis (Sardh *et al,* 2019). Monthly subcutaneous administration of givosiran results in hepatocyte‐specific distribution and downregulation of elevated *ALAS1* mRNA in the liver.

Recently, two new liver‐targeting GalNAc‐siRNA drugs received positive opinion for marketing in Europe, *i.e*. lumasiran and inclisiran (Fitzgerald *et al,* 2017; McGregor *et al,* 2020). Lumasiran targets hydroxyacid oxidase 1 (HAO1) for the treatment of primary hyperoxaluria type 1 (PH1), which is a rare inherited disorder characterised by the overproduction of oxalate. Targeting HAO1 reduces the substrate needed for oxalate production in the liver (McGregor *et al,* 2020). Inclisiran targets the proprotein convertase subtilisin–kexin type 9 (PCSK9) to reduce low‐density lipoprotein (LDL) cholesterol. PCSK9 is a serine protease, which binds to the LDL receptors to induce their lysosomal degradation. Therefore, silencing PCSK9 enhances the half‐life of LDL receptors responsible for cholesterol clearance (Fitzgerald *et al,* 2017). Inclisiran reduces more than 50% LDL cholesterol levels in treated patients with minimal side effects (Khvorova, 2017; Ray *et al,* 2020). Approval of Inclisiran will expand the indications for ONs to not only include rare but also common diseases.

## Concluding comments and future perspectives

In 1978, it was demonstrated that a 13‐mer DNA‐based ON binding to Rous sarcoma virus RNA could inhibit protein expression in cell culture (Zamecnik & Stephenson, 1978), but it was not until 20 years later (1998) that the first ON‐based therapeutic drug fomiversen was approved. By 2016, only two additional drugs (pegaptanib and mipomersen) had been approved (Table 3) but since then the development pace of ON‐based drugs accelerated with 11 ON‐based drugs currently approved (Aartsma‐Rus & Corey, 2020). Yet, many of these drugs display limited efficacy (eteplirsen, golodirsen, viltolersen), and the more efficacious drugs take advantage of local administration (nusinersen). However, GalNAc conjugation and the LNP technology represent delivery breakthroughs that have completely changed the perspective for therapies targeting hepatocytes: in one stroke, this tissue is now accessible for treatment with ONs. These examples of how delivery technologies can be used to overcome delivery hurdles has provided the whole field with a new impetus that will accelerate discoveries for targeting of tissues beyond the liver.

Design and manufacturing of efficient delivery systems is not the only hurdle: the safety of these and their combination with ONs is also paramount. Testing ON safety has not been easy, primarily because many of these drugs have been developed to treat rare diseases. This implies an abundance of preclinical models but limited clinical data. Being a whole new class of drugs, this makes stakeholders wary of missing any step of the development. A striking exception to this is the recent n‐of‐one clinical studies: the development of milasen (Kim *et al,* 2019) was achieved in record time, but it took a high risk/high reward gamble, relying on the safety of IT administration of a chemistry already approved for nusinersen.

A likely leapfrog in the clinical application of therapeutic ONs may come from the results of current clinical trial (NCT04023552; testing APO(a)‐LRx, a GalNAc3‐conjugated ASO) for the lowering of lipoprotein (a) in cardiovascular disease. This trial includes 7,680 patients and the large data set that will be generated is due to change in the landscape for these drugs. By then, many new delivery technologies may have successfully been developed for other targets making this decade the era of ON therapeutics.

**Pending issues**

- A vast array of delivery systems could be used to deliver ONs; however, the majority target the liver or deliver throughout the body without specificity. Further advances are needed to enhance tissue‐specific delivery.
- Our understanding of ON‐mediated toxicities has improved, and many predictive *in vitro* tests have been developed to exclude toxic candidates early in development. Since some toxicities are sequence dependent, it will be important to implement toxicity screening early in the preclinical development of ONs.
- Following the example of milasen, it is anticipated that bespoke ON therapies will be developed for additional brain diseases. A process to guide this development is required.
- Very limited clinical data have been available for many therapeutic ONs, as most target rare disorders and have been tested in dozens or, at best, a few hundred patients. However, this may be about to change thanks to new ON drugs targeting common disorders, such as hyperlipidaemia, which could produce clinical data from thousands of patients and further accelerate development of future therapeutic ONs.

## Conflict of interest

A.A‐R discloses being employed by LUMC which has patents on exon skipping technology. As co‐inventor of some of these patents, AAR is entitled to a share of royalties. AAR further discloses being ad hoc consultant for PTC Therapeutics, Sarepta Therapeutics, Eisa Pharmaceuticals, WaVe Life Sciences, Alpha Anomeric, CRISPR Therapeutics, BioMarin Pharmaceuticals Inc., Global Guidepoint and GLG consultancy, Grunenthal and BioClinica, being a member of the Duchenne Network Steering Committee (BioMarin) and of the scientific advisory boards of ProQR and Philae Pharmaceuticals. Remuneration for these activities is paid to LUMC. LUMC also received speaker honoraria from PTC Therapeutics and BioMarin Pharmaceuticals. A.G and R.W.J.C are inventors of several patents describing the use of antisense oligonucleotides for the treatment of inherited retinal diseases. C.F is *ad hoc* consultant for Lundbeck Pharma A/S, Valby, DK. W.vR.M discloses being employed by LUMC which has patents on exon skipping technology for brain disorders. As co‐inventor of some of these patents WvRM is entitled to a share of royalties. T.L is a consultant for and has equity interests in Evox Therapeutics Ltd., Oxford, UK. L.E is an employee of SQY therapeutics developing tcDNA antisense oligonucleotides. M.A.D and G.C are co‐inventors of patent WO2016/151523 (RNA interference mediated therapy for neurodegenerative diseases) filed by the University of Trento and are entitled to a share of royalties. S.M.H is an inventor on a patent describing cell‐penetrating peptides and is employed by Oxford Biomedica Plc, Oxford UK. Co‐authors, R.A.M.B, G.G, S.A, L.R.D, S.K, V.A‐G, S.E.B and A.T.G declare no conflict of interests.

## For more information

- www.clinicaltrials.gov
- https://www.ich.org/
- https://www.ema.europa.eu
- https://www.fda.gov
- https://antisenserna.eu/

## References

[emmm202013243-bib-0001] Aartsma‐Rus A (2017) FDA approval of nusinersen for spinal muscular atrophy makes 2016 the year of splice modulating oligonucleotides. Nucleic Acid Ther 27: 67–69 28346110 10.1089/nat.2017.0665

[emmm202013243-bib-0002] Aartsma‐Rus A , Arechavala‐Gomeza V (2018) Why dystrophin quantification is key in the eteplirsen saga. Nat Rev Neurol 14: 454–456 29967362 10.1038/s41582-018-0033-8

[emmm202013243-bib-0003] Aartsma‐Rus A , Corey DR (2020) The 10th oligonucleotide therapy approved: golodirsen for duchenne muscular dystrophy. Nucleic Acid Ther 30: 67–70 32043902 10.1089/nat.2020.0845 PMC7133412

[emmm202013243-bib-0004] Achberger K , Probst C , Haderspeck J , Bolz S , Rogal J , Chuchuy J , Nikolova M , Cora V , Antkowiak L , Haq W *et al* (2019) Merging organoid and organ‐on‐a‐chip technology to generate complex multi‐layer tissue models in a human retina‐on‐a‐chip platform. Elife 8: e46188 31451149 10.7554/eLife.46188 PMC6777939

[emmm202013243-bib-0005] Adams D , Gonzalez‐Duarte A , O'Riordan WD , Yang CC , Ueda M , Kristen AV , Tournev I , Schmidt HH , Coelho T , Berk JL *et al* (2018) Patisiran, an RNAi therapeutic, for hereditary transthyretin amyloidosis. N Engl J Med 379: 11–21 29972753 10.1056/NEJMoa1716153

[emmm202013243-bib-0008] Agrawal S , Kandimalla ER (2004) Role of Toll‐like receptors in antisense and siRNA [corrected]. Nat Biotechnol 22: 1533–1537 15583662 10.1038/nbt1042

[emmm202013243-bib-0009] Akinc A , Querbes W , De S , Qin J , Frank‐Kamenetsky M , Jayaprakash KN , Jayaraman M , Rajeev KG , Cantley WL , Dorkin JR *et al* (2010) Targeted delivery of RNAi therapeutics with endogenous and exogenous ligand‐based mechanisms. Mol Ther 18: 1357–1364 20461061 10.1038/mt.2010.85 PMC2911264

[emmm202013243-bib-0010] Akinc A , Zumbuehl A , Goldberg M , Leshchiner ES , Busini V , Hossain N , Bacallado SA , Nguyen DN , Fuller J , Alvarez R *et al* (2008) A combinatorial library of lipid‐like materials for delivery of RNAi therapeutics. Nat Biotechnol 26: 561–569 18438401 10.1038/nbt1402 PMC3014085

[emmm202013243-bib-0011] Alvarez‐Erviti L , Seow Y , Yin H , Betts C , Lakhal S , Wood MJ (2011) Delivery of siRNA to the mouse brain by systemic injection of targeted exosomes. Nat Biotechnol 29: 341–345 21423189 10.1038/nbt.1807

[emmm202013243-bib-0012] Ammala C , Drury 3rd WJ , Knerr L , Ahlstedt I , Stillemark‐Billton P , Wennberg‐Huldt C , Andersson EM , Valeur E , Jansson‐Lofmark R , Janzen D *et al* (2018) Targeted delivery of antisense oligonucleotides to pancreatic beta‐cells. Science Adv 4: eaat3386 10.1126/sciadv.aat3386 PMC6192685 30345352

[emmm202013243-bib-0013] Andersson P , den Besten C (2019) CHAPTER 20 preclinical and clinical drug‐metabolism, pharmacokinetics and safety of therapeutic oligonucleotides. In Advances in nucleic acid therapeutics, Agrawal S , Gait MJ (eds), pp 474–531. London: The Royal Society of Chemistry

[emmm202013243-bib-0014] Apter S , Hertz M , Rubinstein ZJ , Zissin R (1990) Gossypiboma in the early post‐operative period: a diagnostic problem. Clin Radiol 42: 128–129 2394069 10.1016/s0009-9260(05)82084-7

[emmm202013243-bib-0015] Arechavala‐Gomeza V , Khoo B , Aartsma‐Rus A (2014) Splicing modulation therapy in the treatment of genetic diseases. Appl Clin Genet 7: 245–252 25506237 10.2147/TACG.S71506 PMC4259397

[emmm202013243-bib-0016] Aupy P , Echevarria L , Relizani K , Zarrouki F , Haeberli A , Komisarski M , Tensorer T , Jouvion G , Svinartchouk F , Garcia L *et al* (2020) Identifying and avoiding tcDNA‐ASO sequence‐specific toxicity for the development of DMD exon 51 skipping therapy. Mol Ther Nucleic Acids 19: 371–383 31881528 10.1016/j.omtn.2019.11.020 PMC7063478

[emmm202013243-bib-0017] Barchet W , Wimmenauer V , Schlee M , Hartmann G (2008) Accessing the therapeutic potential of immunostimulatory nucleic acids. Curr Opin Immunol 20: 389–395 18652893 10.1016/j.coi.2008.07.007

[emmm202013243-bib-0018] Barile L , Vassalli G (2017) Exosomes: Therapy delivery tools and biomarkers of diseases. Pharmacol Ther 174: 63–78 28202367 10.1016/j.pharmthera.2017.02.020

[emmm202013243-bib-0019] Bennett CF , Baker BF , Pham N , Swayze E , Geary RS (2017) Pharmacology of antisense drugs. Annu Rev Pharmacol Toxicol 57: 81–105 27732800 10.1146/annurev-pharmtox-010716-104846

[emmm202013243-bib-0020] Benson MD , Waddington‐Cruz M , Berk JL , Polydefkis M , Dyck PJ , Wang AK , Plante‐Bordeneuve V , Barroso FA , Merlini G , Obici L *et al* (2018) Inotersen treatment for patients with hereditary transthyretin amyloidosis. N Engl J Med 379: 22–31 29972757 10.1056/NEJMoa1716793 PMC12611561

[emmm202013243-bib-0021] Betts CA , McClorey G , Healicon R , Hammond SM , Manzano R , Muses S , Ball V , Godfrey C , Merritt TM , van Westering T *et al* (2019) Cmah‐dystrophin deficient mdx mice display an accelerated cardiac phenotype that is improved following peptide‐PMO exon skipping treatment. Hum Mol Genet 28: 396–406 30281092 10.1093/hmg/ddy346 PMC6337703

[emmm202013243-bib-0022] Biscans A , Caiazzi J , Davis S , McHugh N , Sousa J , Khvorova A (2020) The chemical structure and phosphorothioate content of hydrophobically modified siRNAs impact extrahepatic distribution and efficacy. Nucleic Acids Res 48: 7665–7680 32672813 10.1093/nar/gkaa595 PMC7430635

[emmm202013243-bib-0023] Boisguerin P , Deshayes S , Gait MJ , O'Donovan L , Godfrey C , Betts CA , Wood MJ , Lebleu B (2015) Delivery of therapeutic oligonucleotides with cell penetrating peptides. Adv Drug Deliv Rev 87: 52–67 25747758 10.1016/j.addr.2015.02.008 PMC7102600

[emmm202013243-bib-0024] Bosgra S , Sipkens J , de Kimpe S , den Besten C , Datson N , van Deutekom J (2019) The pharmacokinetics of 2'‐O‐methyl phosphorothioate antisense oligonucleotides: experiences from developing exon Skipping therapies for duchenne muscular dystrophy. Nucleic Acid Ther 29: 305–322 31429628 10.1089/nat.2019.0805

[emmm202013243-bib-0025] Buijsen RAM , Toonen LJA , Gardiner SL , van Roon‐Mom WMC (2019) Genetics, mechanisms, and therapeutic progress in polyglutamine spinocerebellar ataxias. Neurotherapeutics 16: 263–286 30607747 10.1007/s13311-018-00696-y PMC6554265

[emmm202013243-bib-0026] Burdick AD , Sciabola S , Mantena SR , Hollingshead BD , Stanton R , Warneke JA , Zeng M , Martsen E , Medvedev A , Makarov SS *et al* (2014) Sequence motifs associated with hepatotoxicity of locked nucleic acid–modified antisense oligonucleotides. Nucleic Acids Res 42: 4882–4891 24550163 10.1093/nar/gku142 PMC4005641

[emmm202013243-bib-0027] Burel SA , Hart CE , Cauntay P , Hsiao J , Machemer T , Katz M , Watt A , Bui HH , Younis H , Sabripour M *et al* (2016) Hepatotoxicity of high affinity gapmer antisense oligonucleotides is mediated by RNase H1 dependent promiscuous reduction of very long pre‐mRNA transcripts. Nucleic Acids Res 44: 2093–2109 26553810 10.1093/nar/gkv1210 PMC4797265

[emmm202013243-bib-0028] Burrer R , Neuman BW , Ting JP , Stein DA , Moulton HM , Iversen PL , Kuhn P , Buchmeier MJ (2007) Antiviral effects of antisense morpholino oligomers in murine coronavirus infection models. J Virol 81: 5637–5648 17344287 10.1128/JVI.02360-06 PMC1900280

[emmm202013243-bib-0029] Catuogno S , Esposito CL , De Franciscis V (2016) Aptamer‐mediated targeted delivery of therapeutics: an update. Pharmaceuticals 9: 69 10.3390/ph9040069 PMC5198044 27827876

[emmm202013243-bib-0030] Catuogno S , Rienzo A , Di Vito A , Esposito CL , de Franciscis V (2015) Selective delivery of therapeutic single strand antimiRs by aptamer‐based conjugates. J Control Release 210: 147–159 25998051 10.1016/j.jconrel.2015.05.276

[emmm202013243-bib-0031] Cha W , Fan R , Miao Y , Zhou Y , Qin C , Shan X , Wan X , Li J (2017) Mesoporous silica nanoparticles as carriers for intracellular delivery of nucleic acids and subsequent therapeutic applications. Molecules 22: 782 10.3390/molecules22050782 PMC6154527 28492505

[emmm202013243-bib-0032] Chen S , Tam YYC , Lin PJC , Sung MMH , Tam YK , Cullis PR (2016) Influence of particle size on the in vivo potency of lipid nanoparticle formulations of siRNA. J Control Release 235: 236–244 27238441 10.1016/j.jconrel.2016.05.059

[emmm202013243-bib-0033] Chi X , Gatti P , Papoian T (2017) Safety of antisense oligonucleotide and siRNA‐based therapeutics. Drug Discov Today 22: 823–833 28159625 10.1016/j.drudis.2017.01.013

[emmm202013243-bib-0034] Chow MYT , Qiu Y , Lam JKW (2020) Inhaled RNA therapy: from promise to reality. Trends Pharmacol Sci 41: 715–729 32893004 10.1016/j.tips.2020.08.002 PMC7471058

[emmm202013243-bib-0035] Cideciyan AV , Jacobson SG , Drack AV , Ho AC , Charng J , Garafalo AV , Roman AJ , Sumaroka A , Han IC , Hochstedler MD *et al* (2019) Effect of an intravitreal antisense oligonucleotide on vision in Leber congenital amaurosis due to a photoreceptor cilium defect. Nat Med 25: 225–228 30559420 10.1038/s41591-018-0295-0

[emmm202013243-bib-0036] Cirak S , Arechavala‐Gomeza V , Guglieri M , Feng L , Torelli S , Anthony K , Abbs S , Garralda ME , Bourke J , Wells DJ *et al* (2011) Exon skipping and dystrophin restoration in patients with Duchenne muscular dystrophy after systemic phosphorodiamidate morpholino oligomer treatment: an open‐label, phase 2, dose‐escalation study. Lancet 378: 595–605 21784508 10.1016/S0140-6736(11)60756-3 PMC3156980

[emmm202013243-bib-0037] Cleal K , He L , Watson PD , Jones AT (2013) Endocytosis, intracellular traffic and fate of cell penetrating peptide based conjugates and nanoparticles. Curr Pharm Des 19: 2878–2894 23140451 10.2174/13816128113199990297

[emmm202013243-bib-0038] Collin RW , den Hollander AI , van der Velde‐Visser SD , Bennicelli J , Bennett J , Cremers FP (2012) Antisense oligonucleotide (AON)‐based therapy for leber congenital amaurosis caused by a frequent mutation in CEP290. Mol Ther Nucleic Acids 1: e14 23343883 10.1038/mtna.2012.3 PMC3381589

[emmm202013243-bib-0039] Crooke ST , Baker BF , Pham NC , Hughes SG , Kwoh TJ , Cai D , Tsimikas S , Geary RS , Bhanot S (2018) The effects of 2'‐O‐methoxyethyl oligonucleotides on renal function in humans. Nucleic Acid Ther 28: 10–22 29185862 10.1089/nat.2017.0693 PMC5790433

[emmm202013243-bib-0040] Crooke ST , Wang S , Vickers TA , Shen W , Liang XH (2017) Cellular uptake and trafficking of antisense oligonucleotides. Nat Biotechnol 35: 230–237 28244996 10.1038/nbt.3779

[emmm202013243-bib-0041] Curtis MA , Kam M , Nannmark U , Anderson MF , Axell MZ , Wikkelso C , Holtas S , van Roon‐Mom WM , Bjork‐Eriksson T , Nordborg C *et al* (2007) Human neuroblasts migrate to the olfactory bulb via a lateral ventricular extension. Science 315: 1243–1249 17303719 10.1126/science.1136281

[emmm202013243-bib-0042] Debacker AJ , Voutila J , Catley M , Blakey D , Habib N (2020) Delivery of oligonucleotides to the liver with GalNAc: from research to registered therapeutic drug. Mol Ther 28: 1759–1771 32592692 10.1016/j.ymthe.2020.06.015 PMC7403466

[emmm202013243-bib-0043] Dhillon S (2020) Viltolarsen: first approval. Drugs 80: 1027–1031 32519222 10.1007/s40265-020-01339-3

[emmm202013243-bib-0044] Dieckmann A , Hagedorn PH , Burki Y , Brugmann C , Berrera M , Ebeling M , Singer T , Schuler F (2018) A sensitive in vitro approach to assess the hybridization‐dependent toxic potential of high affinity gapmer oligonucleotides. Mol Ther Nucleic Acids 10: 45–54 29499955 10.1016/j.omtn.2017.11.004 PMC5725219

[emmm202013243-bib-0045] Ding Y , Jiang Z , Saha K , Kim CS , Kim ST , Landis RF , Rotello VM (2014) Gold nanoparticles for nucleic acid delivery. Mol Ther 22: 1075–1083 24599278 10.1038/mt.2014.30 PMC4048892

[emmm202013243-bib-0046] Dong Y , Love KT , Dorkin JR , Sirirungruang S , Zhang Y , Chen D , Bogorad RL , Yin H , Chen Y , Vegas AJ *et al* (2014) Lipopeptide nanoparticles for potent and selective siRNA delivery in rodents and nonhuman primates. Proc Natl Acad Sci USA 111: 3955–3960 24516150 10.1073/pnas.1322937111 PMC3964096

[emmm202013243-bib-0047] Du L , Kayali R , Bertoni C , Fike F , Hu H , Iversen PL , Gatti RA (2011) Arginine‐rich cell‐penetrating peptide dramatically enhances AMO‐mediated ATM aberrant splicing correction and enables delivery to brain and cerebellum. Hum Mol Genet 20: 3151–3160 21576124 10.1093/hmg/ddr217 PMC3140820

[emmm202013243-bib-0048] Echevarria L , Aupy P , Relizani K , Bestetti T , Griffith G , Blandel F , Komisarski M , Haeberli A , Svinartchouk F , Garcia L *et al* (2019) Evaluating the impact of variable phosphorothioate content in tricyclo‐DNA antisense oligonucleotides in a duchenne muscular dystrophy mouse model. Nucleic Acid Ther 29: 148–160 31009315 10.1089/nat.2018.0773

[emmm202013243-bib-0049] Eckstein F (2014) Phosphorothioates, essential components of therapeutic oligonucleotides. Nucleic Acid Ther 24: 374–387 25353652 10.1089/nat.2014.0506

[emmm202013243-bib-0050] Ellington AD , Szostak JW (1990) In vitro selection of RNA molecules that bind specific ligands. Nature 346: 818–822 1697402 10.1038/346818a0

[emmm202013243-bib-0006] EMA (2012) Refusal of the marketing authorisation for Kynamro (mipomersen). https://www.ema.europa.eu/en/medicines/human/EPAR/kynamro

[emmm202013243-bib-0051] EMA (2016) Assessment Report: Kyndrisa EMA/439369/2016. https://www.ema.europa.eu/en/medicines/human/withdrawn-applications/kyndrisa

[emmm202013243-bib-0007] EMA (2019) Volanesorsen: EU summary of product characteristics. https://www.ema.europa.eu/en/medicines/human/EPAR/waylivra

[emmm202013243-bib-0052] Engelhardt JA , Fant P , Guionaud S , Henry SP , Leach MW , Louden C , Scicchitano MS , Weaver JL , Zabka TS , Frazier KS *et al* (2015) Scientific and regulatory policy committee points‐to‐consider paper*: drug‐induced vascular injury associated with nonsmall molecule therapeutics in preclinical development: part 2. Antisense oligonucleotides. Toxicol Pathol 43: 935–944 25717082 10.1177/0192623315570341

[emmm202013243-bib-0053] Esposito CL , Nuzzo S , Kumar SA , Rienzo A , Lawrence CL , Pallini R , Shaw L , Alder JE , Ricci‐Vitiani L , Catuogno S *et al* (2016) A combined microRNA‐based targeted therapeutic approach to eradicate glioblastoma stem‐like cells. J Control Release 238: 43–57 27448441 10.1016/j.jconrel.2016.07.032

[emmm202013243-bib-0054] FDA (2018) Final Summary Minutes of the Endocrinologic and Metabolic Drugs Advisory Committee Meeting. https://www.fda.gov/media/113979/download

[emmm202013243-bib-0055] Fedorov Y , Anderson EM , Birmingham A , Reynolds A , Karpilow J , Robinson K , Leake D , Marshall WS , Khvorova A (2006) Off‐target effects by siRNA can induce toxic phenotype. RNA 12: 1188–1196 16682561 10.1261/rna.28106 PMC1484448

[emmm202013243-bib-0056] Felgner PL , Gadek TR , Holm M , Roman R , Chan HW , Wenz M , Northrop JP , Ringold GM , Danielsen M (1987) Lipofection: a highly efficient, lipid‐mediated DNA‐transfection procedure. Proc Natl Acad Sci USA 84: 7413–7417 2823261 10.1073/pnas.84.21.7413 PMC299306

[emmm202013243-bib-0057] Finkel RS , Mercuri E , Darras BT , Connolly AM , Kuntz NL , Kirschner J , Chiriboga CA , Saito K , Servais L , Tizzano E *et al* (2017) Nusinersen versus sham control in infantile‐onset spinal muscular atrophy. N Engl J Med 377: 1723–1732 29091570 10.1056/NEJMoa1702752

[emmm202013243-bib-0058] Fire A , Xu S , Montgomery MK , Kostas SA , Driver SE , Mello CC (1998) Potent and specific genetic interference by double‐stranded RNA in *Caenorhabditis elegans* . Nature 391: 806–811 9486653 10.1038/35888

[emmm202013243-bib-0059] Fitzgerald K , White S , Borodovsky A , Bettencourt BR , Strahs A , Clausen V , Wijngaard P , Horton JD , Taubel J , Brooks A *et al* (2017) A Highly durable RNAi therapeutic inhibitor of PCSK9. N Engl J Med 376: 41–51 27959715 10.1056/NEJMoa1609243 PMC5778873

[emmm202013243-bib-0060] Flierl U , Nero TL , Lim B , Arthur JF , Yao Y , Jung SM , Gitz E , Pollitt AY , Zaldivia MT , Jandrot‐Perrus M *et al* (2015) Phosphorothioate backbone modifications of nucleotide‐based drugs are potent platelet activators. J Exp Med 212: 129–137 25646267 10.1084/jem.20140391 PMC4322051

[emmm202013243-bib-0061] Foged C , Nielsen HM (2008) Cell‐penetrating peptides for drug delivery across membrane barriers. Exp Opin Drug Deliv 5: 105–117 10.1517/17425247.5.1.105 18095931

[emmm202013243-bib-0062] Frazier KS (2015) Antisense oligonucleotide therapies: the promise and the challenges from a toxicologic pathologist's perspective. Toxicol Pathol 43: 78–89 25385330 10.1177/0192623314551840

[emmm202013243-bib-0063] Freitag F , Wagner E (2020) Optimizing synthetic nucleic acid and protein nanocarriers: The chemical evolution approach. Adv Drug Deliv Rev 168: 30–54 32246984 10.1016/j.addr.2020.03.005

[emmm202013243-bib-0064] Friedman RC , Farh KK , Burge CB , Bartel DP (2009) Most mammalian mRNAs are conserved targets of microRNAs. Genome Res 19: 92–105 18955434 10.1101/gr.082701.108 PMC2612969

[emmm202013243-bib-0065] Fusser M , Overbye A , Pandya AD , Morch Y , Borgos SE , Kildal W , Snipstad S , Sulheim E , Fleten KG , Askautrud HA *et al* (2019) Cabazitaxel‐loaded Poly(2‐ethylbutyl cyanoacrylate) nanoparticles improve treatment efficacy in a patient derived breast cancer xenograft. J Control Release 293: 183–192 30529259 10.1016/j.jconrel.2018.11.029

[emmm202013243-bib-0066] Garanto A , Chung DC , Duijkers L , Corral‐Serrano JC , Messchaert M , Xiao R , Bennett J , Vandenberghe LH , Collin RW (2016) In vitro and in vivo rescue of aberrant splicing in CEP290‐associated LCA by antisense oligonucleotide delivery. Hum Mol Genet 25: 2552–2563 27106101 10.1093/hmg/ddw118 PMC6086559

[emmm202013243-bib-0067] Garanto A , Duijkers L , Collin RW (2015) Species‐dependent splice recognition of a cryptic exon resulting from a recurrent intronic CEP290 mutation that causes congenital blindness. Int J Mol Sci 16: 5285–5298 25761237 10.3390/ijms16035285 PMC4394476

[emmm202013243-bib-0068] Geary RS (2009) Antisense oligonucleotide pharmacokinetics and metabolism. Expert Opin Drug Metab Toxicol 5: 381–391 19379126 10.1517/17425250902877680

[emmm202013243-bib-0069] Geary RS , Baker BF , Crooke ST (2015) Clinical and preclinical pharmacokinetics and pharmacodynamics of mipomersen (kynamro((R))): a second‐generation antisense oligonucleotide inhibitor of apolipoprotein B. Clin Pharmacokinet 54: 133–146 25559341 10.1007/s40262-014-0224-4 PMC4305106

[emmm202013243-bib-0070] Geary RS , Henry SP , Grillone LR (2002) Fomivirsen: clinical pharmacology and potential drug interactions. Clin Pharmacokinet 41: 255–260 11978144 10.2165/00003088-200241040-00002

[emmm202013243-bib-0071] Geller BL , Li L , Martinez F , Sully E , Sturge CR , Daly SM , Pybus C , Greenberg DE (2018) Morpholino oligomers tested in vitro, in biofilm and in vivo against multidrug‐resistant Klebsiella pneumoniae. J Antimicrob Chemother 73: 1611–1619 29506074 10.1093/jac/dky058 PMC6251509

[emmm202013243-bib-0072] Geller BL , Marshall‐Batty K , Schnell FJ , McKnight MM , Iversen PL , Greenberg DE (2013) Gene‐silencing antisense oligomers inhibit acinetobacter growth in vitro and in vivo. J Infect Dis 208: 1553–1560 24130069 10.1093/infdis/jit460 PMC3805245

[emmm202013243-bib-0073] Gilleron J , Querbes W , Zeigerer A , Borodovsky A , Marsico G , Schubert U , Manygoats K , Seifert S , Andree C , Stoter M *et al* (2013) Image‐based analysis of lipid nanoparticle‐mediated siRNA delivery, intracellular trafficking and endosomal escape. Nat Biotechnol 31: 638–646 23792630 10.1038/nbt.2612

[emmm202013243-bib-0074] Godfrey C , Desviat LR , Smedsrod B , Pietri‐Rouxel F , Denti MA , Disterer P , Lorain S , Nogales‐Gadea G , Sardone V , Anwar R *et al* (2017) Delivery is key: lessons learnt from developing splice‐switching antisense therapies. EMBO Mol Med 9: 545–557 28289078 10.15252/emmm.201607199 PMC5412803

[emmm202013243-bib-0075] Goemans NM , Tulinius M , van den Hauwe M , Kroksmark AK , Buyse G , Wilson RJ , van Deutekom JC , de Kimpe SJ , Lourbakos A , Campion G (2016) Long‐term efficacy, safety, and pharmacokinetics of drisapersen in duchenne muscular dystrophy: results from an open‐label extension study. PLoS One 11: e0161955 27588424 10.1371/journal.pone.0161955 PMC5010191

[emmm202013243-bib-0076] Gonzales CR, Group VISiONCT (2005) Enhanced efficacy associated with early treatment of neovascular age‐related macular degeneration with pegaptanib sodium: an exploratory analysis. Retina 25: 815–827 16205558 10.1097/00006982-200510000-00001

[emmm202013243-bib-0077] Gragoudas ES , Adamis AP , Cunningham ET , Feinsod M , Guyer DR , Group VISiONCT (2004) Pegaptanib for neovascular age‐related macular degeneration. N Engl J Med 351: 2805–2816 15625332 10.1056/NEJMoa042760

[emmm202013243-bib-0078] Graham MJ , Lee RG , Bell 3rd TA , Fu W , Mullick AE , Alexander VJ , Singleton W , Viney N , Geary R , Su J *et al* (2013) Antisense oligonucleotide inhibition of apolipoprotein C‐III reduces plasma triglycerides in rodents, nonhuman primates, and humans. Circ Res 112: 1479–1490 23542898 10.1161/CIRCRESAHA.111.300367

[emmm202013243-bib-0079] Habrant D , Peuziat P , Colombani T , Dallet L , Gehin J , Goudeau E , Evrard B , Lambert O , Haudebourg T , Pitard B (2016) Design of ionizable lipids to overcome the limiting step of endosomal escape: application in the intracellular delivery of mRNA, DNA, and siRNA. J Med Chem 59: 3046–3062 26943260 10.1021/acs.jmedchem.5b01679

[emmm202013243-bib-0080] Hache M , Swoboda KJ , Sethna N , Farrow‐Gillespie A , Khandji A , Xia S , Bishop KM (2016) Intrathecal injections in children with spinal muscular atrophy: nusinersen clinical trial experience. J Child Neurol 31: 899–906 26823478 10.1177/0883073815627882 PMC4871174

[emmm202013243-bib-0081] Hagedorn PH , Hansen BR , Koch T , Lindow M (2017) Managing the sequence‐specificity of antisense oligonucleotides in drug discovery. Nucleic Acids Res 45: 2262–2282 28426096 10.1093/nar/gkx056 PMC5389529

[emmm202013243-bib-0082] Hagedorn PH , Yakimov V , Ottosen S , Kammler S , Nielsen NF , Hog AM , Hedtjarn M , Meldgaard M , Moller MR , Orum H *et al* (2013) Hepatotoxic potential of therapeutic oligonucleotides can be predicted from their sequence and modification pattern. Nucleic Acid Ther 23: 302–310 23952551 10.1089/nat.2013.0436 PMC3760025

[emmm202013243-bib-0083] Hammond SM , Hazell G , Shabanpoor F , Saleh AF , Bowerman M , Sleigh JN , Meijboom KE , Zhou H , Muntoni F , Talbot K *et al* (2016) Systemic peptide‐mediated oligonucleotide therapy improves long‐term survival in spinal muscular atrophy. Proc Natl Acad Sci USA 113: 10962–10967 27621445 10.1073/pnas.1605731113 PMC5047168

[emmm202013243-bib-0084] Hanvey JC , Peffer NJ , Bisi JE , Thomson SA , Cadilla R , Josey JA , Ricca DJ , Hassman CF , Bonham MA , Au KG *et al* (1992) Antisense and antigene properties of peptide nucleic acids. Science 258: 1481–1485 1279811 10.1126/science.1279811

[emmm202013243-bib-0085] Heemskerk HA , de Winter CL , de Kimpe SJ , van Kuik‐Romeijn P , Heuvelmans N , Platenburg GJ , van Ommen GJ , van Deutekom JC , Aartsma‐Rus A (2009) In vivo comparison of 2'‐O‐methyl phosphorothioate and morpholino antisense oligonucleotides for Duchenne muscular dystrophy exon skipping. J Gene Med 11: 257–266 19140108 10.1002/jgm.1288

[emmm202013243-bib-0086] Heemskerk H , de Winter C , van Kuik P , Heuvelmans N , Sabatelli P , Rimessi P , Braghetta P , van Ommen GJ , de Kimpe S , Ferlini A *et al* (2010) Preclinical PK and PD studies on 2'‐O‐methyl‐phosphorothioate RNA antisense oligonucleotides in the mdx mouse model. Mol Ther 18: 1210–1217 20407428 10.1038/mt.2010.72 PMC2889733

[emmm202013243-bib-0087] van der Helm MW , van der Meer AD , Eijkel JC , van den Berg A , Segerink LI (2016) Microfluidic organ‐on‐chip technology for blood‐brain barrier research. Tissue Barriers 4: e1142493 27141422 10.1080/21688370.2016.1142493 PMC4836466

[emmm202013243-bib-0088] Henry SP , Beattie G , Yeh G , Chappel A , Giclas P , Mortari A , Jagels MA , Kornbrust DJ , Levin AA (2002) Complement activation is responsible for acute toxicities in rhesus monkeys treated with a phosphorothioate oligodeoxynucleotide. Int Immunopharmacol 2: 1657–1666 12469940 10.1016/s1567-5769(02)00142-x

[emmm202013243-bib-0089] Henry SP , Giclas PC , Leeds J , Pangburn M , Auletta C , Levin AA , Kornbrust DJ (1997a) Activation of the alternative pathway of complement by a phosphorothioate oligonucleotide: potential mechanism of action. J Pharmacol Exp Ther 281: 810–816 9152389

[emmm202013243-bib-0090] Henry SP , Miner RC , Drew WL , Fitchett J , York‐Defalco C , Rapp LM , Levin AA (2001) Antiviral activity and ocular kinetics of antisense oligonucleotides designed to inhibit CMV replication. Invest Ophthalmol Vis Sci 42: 2646–2651 11581212

[emmm202013243-bib-0091] Henry SP , Narayanan P , Shen L , Bhanot S , Younis HS , Burel SA (2017) Assessment of the effects of 2'‐methoxyethyl antisense oligonucleotides on platelet count in cynomolgus nonhuman primates. Nucleic Acid Ther 27: 197–208 28541820 10.1089/nat.2017.0666

[emmm202013243-bib-0092] Henry SP , Novotny W , Leeds J , Auletta C , Kornbrust DJ (1997b) Inhibition of coagulation by a phosphorothioate oligonucleotide. Antisense Nucleic Acid Drug Dev 7: 503–510 9361909 10.1089/oli.1.1997.7.503

[emmm202013243-bib-0093] Henry SP , Seguin R , Cavagnaro J , Berman C , Tepper J , Kornbrust D (2016) Considerations for the characterization and interpretation of results related to alternative complement activation in monkeys associated with oligonucleotide‐based therapeutics. Nucleic Acid Ther 26: 210–215 26981618 10.1089/nat.2015.0593

[emmm202013243-bib-0094] Heo YA (2020) Golodirsen: first approval. Drugs 80: 329–333 32026421 10.1007/s40265-020-01267-2

[emmm202013243-bib-0095] den Hollander AI , Koenekoop RK , Yzer S , Lopez I , Arends ML , Voesenek KE , Zonneveld MN , Strom TM , Meitinger T , Brunner HG *et al* (2006) Mutations in the CEP290 (NPHP6) gene are a frequent cause of Leber congenital amaurosis. Am J Hum Genet 79: 556–561 16909394 10.1086/507318 PMC1559533

[emmm202013243-bib-0096] Hsu WH , Csaba N , Alexander C , Garcia‐Fuentes M (2020) Polyphosphazenes for the delivery of biopharmaceuticals. J Appl Polym Sci 137: 48688

[emmm202013243-bib-0097] Huh D , Matthews BD , Mammoto A , Montoya‐Zavala M , Hsin HY , Ingber DE (2010) Reconstituting organ‐level lung functions on a chip. Science 328: 1662–1668 20576885 10.1126/science.1188302 PMC8335790

[emmm202013243-bib-0098] Jaax ME , Krauel K , Marschall T , Brandt S , Gansler J , Furll B , Appel B , Fischer S , Block S , Helm CA *et al* (2013) Complex formation with nucleic acids and aptamers alters the antigenic properties of platelet factor 4. Blood 122: 272–281 23673861 10.1182/blood-2013-01-478966 PMC3709655

[emmm202013243-bib-0099] Janas MM , Schlegel MK , Harbison CE , Yilmaz VO , Jiang Y , Parmar R , Zlatev I , Castoreno A , Xu H , Shulga‐Morskaya S *et al* (2018) Selection of GalNAc‐conjugated siRNAs with limited off‐target‐driven rat hepatotoxicity. Nat Commun 9: 723 29459660 10.1038/s41467-018-02989-4 PMC5818625

[emmm202013243-bib-0100] Jarver P , O'Donovan L , Gait MJ (2014) A chemical view of oligonucleotides for exon skipping and related drug applications. Nucleic Acid Ther 24: 37–47 24171481 10.1089/nat.2013.0454 PMC3923385

[emmm202013243-bib-0101] Jayaraman M , Ansell SM , Mui BL , Tam YK , Chen J , Du X , Butler D , Eltepu L , Matsuda S , Narayanannair JK *et al* (2012) Maximizing the potency of siRNA lipid nanoparticles for hepatic gene silencing in vivo. Angew Chem 51: 8529–8533 22782619 10.1002/anie.201203263 PMC3470698

[emmm202013243-bib-0102] Jayasena SD (1999) Aptamers: an emerging class of molecules that rival antibodies in diagnostics. Clin Chem 45: 1628–1650 10471678

[emmm202013243-bib-0103] Jeppesen DK , Fenix AM , Franklin JL , Higginbotham JN , Zhang Q , Zimmerman LJ , Liebler DC , Ping J , Liu Q , Evans R *et al* (2019) Reassessment of exosome composition. Cell 177: 428–445.e18 30951670 10.1016/j.cell.2019.02.029 PMC6664447

[emmm202013243-bib-0104] Judge AD , Bola G , Lee AC , MacLachlan I (2006) Design of noninflammatory synthetic siRNA mediating potent gene silencing in vivo. Mol Ther 13: 494–505 16343994 10.1016/j.ymthe.2005.11.002

[emmm202013243-bib-0105] Juliano RL , Wang L , Tavares F , Brown EG , James L , Ariyarathna Y , Ming X , Mao C , Suto M (2018) Structure‐activity relationships and cellular mechanism of action of small molecules that enhance the delivery of oligonucleotides. Nucleic Acids Res 46: 1601–1613 29361039 10.1093/nar/gkx1320 PMC5829638

[emmm202013243-bib-0106] Khvorova A (2017) Oligonucleotide therapeutics ‐ a new class of cholesterol‐lowering drugs. N Engl J Med 376: 4–7 28052224 10.1056/NEJMp1614154

[emmm202013243-bib-0107] Kim HJ , Huh D , Hamilton G , Ingber DE (2012) Human gut‐on‐a‐chip inhabited by microbial flora that experiences intestinal peristalsis‐like motions and flow. Lab Chip 12: 2165–2174 22434367 10.1039/c2lc40074j

[emmm202013243-bib-0108] Kim J , Hu C , Moufawad El Achkar C , Black LE , Douville J , Larson A , Pendergast MK , Goldkind SF , Lee EA , Kuniholm A *et al* (2019) Patient‐customized oligonucleotide therapy for a rare genetic disease. N Engl J Med 381: 1644–1652 31597037 10.1056/NEJMoa1813279 PMC6961983

[emmm202013243-bib-0109] Klein AF , Varela MA , Arandel L , Holland A , Naouar N , Arzumanov A , Seoane D , Revillod L , Bassez G , Ferry A *et al* (2019) Peptide‐conjugated oligonucleotides evoke long‐lasting myotonic dystrophy correction in patient‐derived cells and mice. J Clin Invest 129: 4739–4744 31479430 10.1172/JCI128205 PMC6819114

[emmm202013243-bib-0110] Kline JN , Krieg AM (2008) Toll‐like receptor 9 activation with CpG oligodeoxynucleotides for asthma therapy. Drug News Perspect 21: 434–439 19034349 10.1358/dnp.2008.21.8.1272133

[emmm202013243-bib-0111] Koshkin AA , Singh SK , Nielsen P , Rajwanshi VK , Kumar R , Meldgaard M , Olsen CE , Wengel J (1998) LNA (Locked Nucleic Acids): synthesis of the adenine, cytosine, guanine, 5‐methylcytosine, thymine and uracil bicyclonucleoside monomers, oligomerisation, and unprecedented nucleic acid recognition. Tetrahedron 54: 3607–3630

[emmm202013243-bib-0112] Kozlu S , Caban S , Yerlikaya F , Fernandez‐Megia E , Novoa‐Carballal R , Riguera R , Yemisci M , Gursoy‐Ozdemir Y , Dalkara T , Couvreur P *et al* (2014) An aquaporin 4 antisense oligonucleotide loaded, brain targeted nanoparticulate system design. Pharmazie 69: 340–345 24855824

[emmm202013243-bib-0113] Krieg AM (1998) The CpG motif: implications for clinical immunology. BioDrugs 10: 341–346 18020606 10.2165/00063030-199810050-00001

[emmm202013243-bib-0114] Krieg AM (2006) Therapeutic potential of Toll‐like receptor 9 activation. Nat Rev Drug Discov 5: 471–484 16763660 10.1038/nrd2059

[emmm202013243-bib-0115] Krieg AM (2007) Antiinfective applications of toll‐like receptor 9 agonists. Proc Am Thorac Soc 4: 289–294 17607015 10.1513/pats.200701-021AW PMC2647632

[emmm202013243-bib-0116] Krieg AM , Davis HL (2001) Enhancing vaccines with immune stimulatory CpG DNA. Curr Opin Mol Ther 3: 15–24 11249727

[emmm202013243-bib-0117] Kulkarni JA , Witzigmann D , Leung J , van der Meel R , Zaifman J , Darjuan MM , Grisch‐Chan HM , Thony B , Tam YYC , Cullis PR (2019) Fusion‐dependent formation of lipid nanoparticles containing macromolecular payloads. Nanoscale 11: 9023–9031 31021343 10.1039/c9nr02004g

[emmm202013243-bib-0118] Lankveld DP , Van Loveren H , Baken KA , Vandebriel RJ (2010) In vitro testing for direct immunotoxicity: state of the art. Methods Mol Biol 598: 401–423 19967527 10.1007/978-1-60761-401-2_26

[emmm202013243-bib-0119] Lee HJ , Boado RJ , Braasch DA , Corey DR , Pardridge WM (2002) Imaging gene expression in the brain in vivo in a transgenic mouse model of Huntington's disease with an antisense radiopharmaceutical and drug‐targeting technology. J Nucl Med 43: 948–956 12097468

[emmm202013243-bib-0120] Lee H , Kim DS , Ha SK , Choi I , Lee JM , Sung JH (2017) A pumpless multi‐organ‐on‐a‐chip (MOC) combined with a pharmacokinetic‐pharmacodynamic (PK‐PD) model. Biotechnol Bioeng 114: 432–443 27570096 10.1002/bit.26087

[emmm202013243-bib-0121] Lee KY , Mooney DJ (2012) Alginate: properties and biomedical applications. Prog Polym Sci 37: 106–126 22125349 10.1016/j.progpolymsci.2011.06.003 PMC3223967

[emmm202013243-bib-0122] Lee RT , Lin P , Lee YC (1984) New synthetic cluster ligands for galactose/N‐acetylgalactosamine‐specific lectin of mammalian liver. Biochemistry 23: 4255–4261 6487600 10.1021/bi00313a037

[emmm202013243-bib-0123] Lehto T , Ezzat K , Wood MJA , El Andaloussi S (2016) Peptides for nucleic acid delivery. Adv Drug Deliv Rev 106: 172–182 27349594 10.1016/j.addr.2016.06.008

[emmm202013243-bib-0124] Levin AA , Henry SP (2008) Toxicology of oligonucleotide therapeutics and understanding the relevance of the toxicities. In Preclinical safety evaluation of biopharmaceuticals: a science‐based approach to facilitating clinical trials, Cavagnaro JA (ed), pp 537–574. Hoboken, NJ: John Wiley & Sons Inc

[emmm202013243-bib-0125] Li JY , Ren YP , Yuan Y , Ji SM , Zhou SP , Wang LJ , Mou ZZ , Li L , Lu W , Zhou TY (2016) Preclinical PK/PD model for combined administration of erlotinib and sunitinib in the treatment of A549 human NSCLC xenograft mice. Acta Pharmacol Sin 37: 930–940 27180983 10.1038/aps.2016.55 PMC4933764

[emmm202013243-bib-0126] Liang XH , Shen W , Sun H , Kinberger GA , Prakash TP , Nichols JG , Crooke ST (2016a) Hsp90 protein interacts with phosphorothioate oligonucleotides containing hydrophobic 2'‐modifications and enhances antisense activity. Nucleic Acids Res 44: 3892–3907 26945041 10.1093/nar/gkw144 PMC4856991

[emmm202013243-bib-0127] Liang XH , Shen W , Sun H , Migawa MT , Vickers TA , Crooke ST (2016b) Translation efficiency of mRNAs is increased by antisense oligonucleotides targeting upstream open reading frames. Nat Biotechnol 34: 875–880 27398791 10.1038/nbt.3589

[emmm202013243-bib-0128] Liang XH , Sun H , Shen W , Wang S , Yao J , Migawa MT , Bui HH , Damle SS , Riney S , Graham MJ *et al* (2017) Antisense oligonucleotides targeting translation inhibitory elements in 5' UTRs can selectively increase protein levels. Nucleic Acids Res 45: 9528–9546 28934489 10.1093/nar/gkx632 PMC5766168

[emmm202013243-bib-0129] Lindow M , Vornlocher HP , Riley D , Kornbrust DJ , Burchard J , Whiteley LO , Kamens J , Thompson JD , Nochur S , Younis H *et al* (2012) Assessing unintended hybridization‐induced biological effects of oligonucleotides. Nat Biotechnol 30: 920–923 23051805 10.1038/nbt.2376

[emmm202013243-bib-0130] Malmsten M (2013) Inorganic nanomaterials as delivery systems for proteins, peptides, DNA, and siRNA. Curr Opin Colloid Interface Sci 18: 468–480

[emmm202013243-bib-0131] Marwick C (1998) First "antisense" drug will treat CMV retinitis. JAMA 280: 871 9739955

[emmm202013243-bib-0132] Mathew V , Wang AK (2019) Inotersen: new promise for the treatment of hereditary transthyretin amyloidosis. Drug Design Dev Ther 13: 1515–1525 10.2147/DDDT.S162913 PMC6507904 31118583

[emmm202013243-bib-0133] Matsuda S , Keiser K , Nair JK , Charisse K , Manoharan RM , Kretschmer P , Peng CG , Kelin AV , Kandasamy P , Willoughby JL *et al* (2015) siRNA conjugates carrying sequentially assembled trivalent N‐acetylgalactosamine linked through nucleosides elicit robust gene silencing in vivo in hepatocytes. ACS Chem Biol 10: 1181–1187 25730476 10.1021/cb501028c

[emmm202013243-bib-0134] McAleer CW , Pointon A , Long CJ , Brighton RL , Wilkin BD , Bridges LR , Narasimhan Sriram N , Fabre K , McDougall R , Muse VP *et al* (2019) On the potential of in vitro organ‐chip models to define temporal pharmacokinetic‐pharmacodynamic relationships. Sci Rep 9: 9619 31270362 10.1038/s41598-019-45656-4 PMC6610665

[emmm202013243-bib-0135] McGregor TL , Hunt KA , Yee E , Mason D , Nioi P , Ticau S , Pelosi M , Loken PR , Finer S , Lawlor DA *et al* (2020) Characterising a healthy adult with a rare HAO1 knockout to support a therapeutic strategy for primary hyperoxaluria. Elife 9: e54363 32207686 10.7554/eLife.54363 PMC7108859

[emmm202013243-bib-0136] McNamara 2nd JO , Andrechek ER , Wang Y , Viles KD , Rempel RE , Gilboa E , Sullenger BA , Giangrande PH (2006) Cell type‐specific delivery of siRNAs with aptamer‐siRNA chimeras. Nat Biotechnol 24: 1005–1015 16823371 10.1038/nbt1223

[emmm202013243-bib-0137] van der Meer AD , van den Berg A (2012) Organs‐on‐chips: breaking the in vitro impasse. Integr Biol 4: 461–470 10.1039/c2ib00176d 22388577

[emmm202013243-bib-0138] Mercuri E , Darras BT , Chiriboga CA , Day JW , Campbell C , Connolly AM , Iannaccone ST , Kirschner J , Kuntz NL , Saito K *et al* (2018) Nusinersen versus sham control in later‐onset spinal muscular atrophy. N Engl J Med 378: 625–635 29443664 10.1056/NEJMoa1710504

[emmm202013243-bib-0139] Mignani S , Shi X , Zablocka M , Majoral JP (2019) Dendrimer‐enabled therapeutic antisense delivery systems as innovation in medicine. Bioconjug Chem 30: 1938–1950 31246431 10.1021/acs.bioconjchem.9b00385

[emmm202013243-bib-0140] Miller JW , Urbinati CR , Teng‐Umnuay P , Stenberg MG , Byrne BJ , Thornton CA , Swanson MS (2000) Recruitment of human muscleblind proteins to (CUG)(n) expansions associated with myotonic dystrophy. EMBO J 19: 4439–4448 10970838 10.1093/emboj/19.17.4439 PMC302046

[emmm202013243-bib-0141] Mitrpant C , Adams AM , Meloni PL , Muntoni F , Fletcher S , Wilton SD (2009) Rational design of antisense oligomers to induce dystrophin exon skipping. Mol Ther 17: 1418–1426 19293776 10.1038/mt.2009.49 PMC2835229

[emmm202013243-bib-0142] Moisan A , Gubler M , Zhang JD , Tessier Y , Dumong Erichsen K , Sewing S , Gerard R , Avignon B , Huber S , Benmansour F *et al* (2017) Inhibition of EGF uptake by nephrotoxic antisense drugs in vitro and implications for preclinical safety profiling. Mol Ther Nucleic Acids 6: 89–105 28325303 10.1016/j.omtn.2016.11.006 PMC5363415

[emmm202013243-bib-0143] Monia BP , Lesnik EA , Gonzalez C , Lima WF , McGee D , Guinosso CJ , Kawasaki AM , Cook PD , Freier SM (1993) Evaluation of 2'‐modified oligonucleotides containing 2'‐deoxy gaps as antisense inhibitors of gene expression. J Biol Chem 268: 14514–14522 8390996

[emmm202013243-bib-0144] Monteith DK , Henry SP , Howard RB , Flournoy S , Levin AA , Bennett CF , Crooke ST (1997) Immune stimulation–a class effect of phosphorothioate oligodeoxynucleotides in rodents. Anticancer Drug Des 12: 421–432 9236857

[emmm202013243-bib-0145] Morgan E , Wupperfeld D , Morales D , Reich N (2019) Shape matters: gold nanoparticle shape impacts the biological activity of siRNA delivery. Bioconjug Chem 30: 853–860 30735028 10.1021/acs.bioconjchem.9b00004

[emmm202013243-bib-0146] Muller TD , Finan B , Bloom SR , D'Alessio D , Drucker DJ , Flatt PR , Fritsche A , Gribble F , Grill HJ , Habener JF *et al* (2019) Glucagon‐like peptide 1 (GLP‐1). Mol Metab 30: 72–130 31767182 10.1016/j.molmet.2019.09.010 PMC6812410

[emmm202013243-bib-0147] Narayanan P , Shen L , Curtis BR , Bourdon MA , Nolan JP , Gupta S , Hoffmaster C , Zhou F , Christian B , Schaubhut JL *et al* (2018) Investigation into the mechanism(s) that leads to platelet decreases in Cynomolgus monkeys during administration of ISIS 104838, a 2'‐MOE‐modified antisense oligonucleotide. Toxicol Sci 164: 613–626 29846725 10.1093/toxsci/kfy119

[emmm202013243-bib-0148] Negus SS , Banks ML (2018) Pharmacokinetic‐pharmacodynamic (PKPD) analysis with drug discrimination. Curr Top Behav Neurosci 39: 245–259 27571746 10.1007/7854_2016_36 PMC5446801

[emmm202013243-bib-0149] Ng EW , Shima DT , Calias P , Cunningham Jr ET , Guyer DR , Adamis AP (2006) Pegaptanib, a targeted anti‐VEGF aptamer for ocular vascular disease. Nat Rev Drug Discov 5: 123–132 16518379 10.1038/nrd1955

[emmm202013243-bib-0150] Nielsen PE , Egholm M , Berg RH , Buchardt O (1991) Sequence‐selective recognition of DNA by strand displacement with a thymine‐substituted polyamide. Science 254: 1497–1500 1962210 10.1126/science.1962210

[emmm202013243-bib-0151] Obika S , Nanbu D , Hari Y , Andoh J‐I , Morio K‐I , Doi T , Imanishi T (1998) Stability and structural features of the duplexes containing nucleoside analogues with a fixed N‐type conformation, 2′‐O, 4′‐C‐methyleneribonucleosides. Tetrahedron Lett 39: 5401–5404

[emmm202013243-bib-0152] Osborn MF , Coles AH , Biscans A , Haraszti RA , Roux L , Davis S , Ly S , Echeverria D , Hassler MR , Godinho B *et al* (2019) Hydrophobicity drives the systemic distribution of lipid‐conjugated siRNAs via lipid transport pathways. Nucleic Acids Res 47: 1070–1081 30535404 10.1093/nar/gky1232 PMC6379714

[emmm202013243-bib-0153] Pardridge WM (2007) Blood‐brain barrier delivery. Drug Discov Today 12: 54–61 17198973 10.1016/j.drudis.2006.10.013

[emmm202013243-bib-0154] Parfitt DA , Lane A , Ramsden CM , Carr AJ , Munro PM , Jovanovic K , Schwarz N , Kanuga N , Muthiah MN , Hull S *et al* (2016) Identification and correction of mechanisms underlying inherited blindness in human iPSC‐derived optic cups. Cell Stem Cell 18: 769–781 27151457 10.1016/j.stem.2016.03.021 PMC4899423

[emmm202013243-bib-0155] Pathan M , Fonseka P , Chitti SV , Kang T , Sanwlani R , Van Deun J , Hendrix A , Mathivanan S (2019) Vesiclepedia 2019: a compendium of RNA, proteins, lipids and metabolites in extracellular vesicles. Nucleic Acids Res 47: D516–D519 30395310 10.1093/nar/gky1029 PMC6323905

[emmm202013243-bib-0156] Peng Y , Zhu X , Qiu L (2016) Electroneutral composite polymersomes self‐assembled by amphiphilic polyphosphazenes for effective miR‐200c in vivo delivery to inhibit drug resistant lung cancer. Biomaterials 106: 1–12 27541441 10.1016/j.biomaterials.2016.08.001

[emmm202013243-bib-0157] Penichet ML , Kang YS , Pardridge WM , Morrison SL , Shin SU (1999) An antibody‐avidin fusion protein specific for the transferrin receptor serves as a delivery vehicle for effective brain targeting: initial applications in anti‐HIV antisense drug delivery to the brain. J Immunol 163: 4421–4426 10510383

[emmm202013243-bib-0158] Pooga M , Langel U (2015) Classes of Cell‐Penetrating Peptides. Methods Mol Biol 1324: 3–28 26202259 10.1007/978-1-4939-2806-4_1

[emmm202013243-bib-0159] Prakash TP , Mullick AE , Lee RG , Yu J , Yeh ST , Low A , Chappell AE , Ostergaard ME , Murray S , Gaus HJ *et al* (2019) Fatty acid conjugation enhances potency of antisense oligonucleotides in muscle. Nucleic Acids Res 47: 6029–6044 31127296 10.1093/nar/gkz354 PMC6614804

[emmm202013243-bib-0160] Ran FA , Hsu PD , Wright J , Agarwala V , Scott DA , Zhang F (2013) Genome engineering using the CRISPR‐Cas9 system. Nat Protoc 8: 2281–2308 24157548 10.1038/nprot.2013.143 PMC3969860

[emmm202013243-bib-0161] Ray KK , Wright RS , Kallend D , Koenig W , Leiter LA , Raal FJ , Bisch JA , Richardson T , Jaros M , Wijngaard PLJ *et al* (2020) Two phase 3 trials of inclisiran in patients with elevated LDL cholesterol. N Engl J Med 382: 1507–1519 32187462 10.1056/NEJMoa1912387

[emmm202013243-bib-0162] Renneberg D , Leumann CJ (2002) Watson‐Crick base‐pairing properties of tricyclo‐DNA. J Am Chem Soc 124: 5993–6002 12022832 10.1021/ja025569+

[emmm202013243-bib-0163] Rezvantalab S , Drude NI , Moraveji MK , Guvener N , Koons EK , Shi Y , Lammers T , Kiessling F (2018) PLGA‐based nanoparticles in cancer treatment. Front Pharmacol 9: 1260 30450050 10.3389/fphar.2018.01260 PMC6224484

[emmm202013243-bib-0164] Rivera‐Barahona A , Sanchez‐Alcudia R , Viecelli HM , Rufenacht V , Perez B , Ugarte M , Haberle J , Thony B , Desviat LR (2015) Functional characterization of the spf/ash splicing variation in OTC deficiency of mice and man. PLoS One 10: e0122966 25853564 10.1371/journal.pone.0122966 PMC4390381

[emmm202013243-bib-0165] Roberts TC , Langer R , Wood MJA (2020) Advances in oligonucleotide drug delivery. Nat Rev Drug Discov 19: 673–694 32782413 10.1038/s41573-020-0075-7 PMC7419031

[emmm202013243-bib-0166] Ruckman J , Green LS , Beeson J , Waugh S , Gillette WL , Henninger DD , Claesson‐Welsh L , Janjic N (1998) 2'‐Fluoropyrimidine RNA‐based aptamers to the 165‐amino acid form of vascular endothelial growth factor (VEGF165). Inhibition of receptor binding and VEGF‐induced vascular permeability through interactions requiring the exon 7‐encoded domain. J Biol Chem 273: 20556–20567 9685413 10.1074/jbc.273.32.20556

[emmm202013243-bib-0167] Rudin CM , Holmlund J , Fleming GF , Mani S , Stadler WM , Schumm P , Monia BP , Johnston JF , Geary R , Yu RZ *et al* (2001) Phase I Trial of ISIS 5132, an antisense oligonucleotide inhibitor of c‐raf‐1, administered by 24‐hour weekly infusion to patients with advanced cancer. Clin Cancer Res 7: 1214–1220 11350886

[emmm202013243-bib-0168] Sardh E , Harper P , Balwani M , Stein P , Rees D , Bissell DM , Desnick R , Parker C , Phillips J , Bonkovsky HL *et al* (2019) Phase 1 trial of an RNA interference therapy for acute intermittent porphyria. N Engl J Med 380: 549–558 30726693 10.1056/NEJMoa1807838

[emmm202013243-bib-0169] Schoch KM , Miller TM (2017) Antisense oligonucleotides: translation from mouse models to human neurodegenerative diseases. Neuron 94: 1056–1070 28641106 10.1016/j.neuron.2017.04.010 PMC5821515

[emmm202013243-bib-0170] Schwartz AL , Rup D , Lodish HF (1980) Difficulties in the quantification of asialoglycoprotein receptors on the rat hepatocyte. J Biol Chem 255: 9033–9036 7410410

[emmm202013243-bib-0171] Semple SC , Klimuk SK , Harasym TO , Dos Santos N , Ansell SM , Wong KF , Maurer N , Stark H , Cullis PR , Hope MJ *et al* (2001) Efficient encapsulation of antisense oligonucleotides in lipid vesicles using ionizable aminolipids: formation of novel small multilamellar vesicle structures. Biochim Biophys Acta 1510: 152–166 11342155 10.1016/s0005-2736(00)00343-6

[emmm202013243-bib-0172] Senn JJ , Burel S , Henry SP (2005) Non‐CpG‐containing antisense 2'‐methoxyethyl oligonucleotides activate a proinflammatory response independent of Toll‐like receptor 9 or myeloid differentiation factor 88. J Pharmacol Exp Ther 314: 972–979 15919763 10.1124/jpet.105.084004

[emmm202013243-bib-0173] Seo J , Byun WY , Alisafaei F , Georgescu A , Yi YS , Massaro‐Giordano M , Shenoy VB , Lee V , Bunya VY , Huh D (2019) Multiscale reverse engineering of the human ocular surface. Nat Med 25: 1310–1318 31384041 10.1038/s41591-019-0531-2 PMC6950645

[emmm202013243-bib-0174] Sewing S , Boess F , Moisan A , Bertinetti‐Lapatki C , Minz T , Hedtjaern M , Tessier Y , Schuler F , Singer T , Roth AB (2016) Establishment of a predictive in vitro assay for assessment of the hepatotoxic potential of oligonucleotide drugs. PLoS One 11: e0159431 27442522 10.1371/journal.pone.0159431 PMC4956313

[emmm202013243-bib-0175] Sewing S , Roth AB , Winter M , Dieckmann A , Bertinetti‐Lapatki C , Tessier Y , McGinnis C , Huber S , Koller E , Ploix C *et al* (2017) Assessing single‐stranded oligonucleotide drug‐induced effects in vitro reveals key risk factors for thrombocytopenia. PLoS One 12: e0187574 29107969 10.1371/journal.pone.0187574 PMC5673186

[emmm202013243-bib-0176] Shaffer C (2020) Mist begins to clear for lung delivery of RNA. Nat Biotechnol 38: 1110–1112 33020636 10.1038/s41587-020-0692-z

[emmm202013243-bib-0177] Sheehan JP , Lan HC (1998) Phosphorothioate oligonucleotides inhibit the intrinsic tenase complex. Blood 92: 1617–1625 9716589

[emmm202013243-bib-0178] Shen L , Engelhardt JA , Hung G , Yee J , Kikkawa R , Matson J , Tayefeh B , Machemer T , Giclas PC , Henry SP (2016) Effects of repeated complement activation associated with chronic treatment of cynomolgus monkeys with 2'‐O‐methoxyethyl modified antisense oligonucleotide. Nucleic Acid Ther 26: 236–249 27140858 10.1089/nat.2015.0584

[emmm202013243-bib-0179] Shen W , De Hoyos CL , Migawa MT , Vickers TA , Sun H , Low A , Bell 3rd TA , Rahdar M , Mukhopadhyay S , Hart CE *et al* (2019) Chemical modification of PS‐ASO therapeutics reduces cellular protein‐binding and improves the therapeutic index. Nat Biotechnol 37: 640–650 31036929 10.1038/s41587-019-0106-2

[emmm202013243-bib-0180] Shi B , Keough E , Matter A , Leander K , Young S , Carlini E , Sachs AB , Tao W , Abrams M , Howell B *et al* (2011) Biodistribution of small interfering RNA at the organ and cellular levels after lipid nanoparticle‐mediated delivery. J Histochem Cytochem 59: 727–740 21804077 10.1369/0022155411410885 PMC3261601

[emmm202013243-bib-0181] Shinha K , Nihei W , Ono T , Nakazato R , Kimura H (2020) A pharmacokinetic–pharmacodynamic model based on multi‐organ‐on‐a‐chip for drug–drug interaction studies. Biomicrofluidics 14: 44108 10.1063/5.0011545 PMC8719524 34992705

[emmm202013243-bib-0182] Singh NK , Singh NN , Androphy EJ , Singh RN (2006) Splicing of a critical exon of human Survival Motor Neuron is regulated by a unique silencer element located in the last intron. Mol Cell Biol 26: 1333–1346 16449646 10.1128/MCB.26.4.1333-1346.2006 PMC1367187

[emmm202013243-bib-0183] Smulevitch SV , Simmons CG , Norton JC , Wise TW , Corey DR (1996) Enhancement of strand invasion by oligonucleotides through manipulation of backbone charge. Nat Biotechnol 14: 1700–1704 9634855 10.1038/nbt1296-1700

[emmm202013243-bib-0184] Soldevilla MM , Meraviglia‐Crivelli de Caso D , Menon AP , Pastor F (2018) Aptamer‐iRNAs as therapeutics for cancer treatment. Pharmaceuticals 11: 108 10.3390/ph11040108 PMC6315413 30340426

[emmm202013243-bib-0185] Song E , Zhu P , Lee SK , Chowdhury D , Kussman S , Dykxhoorn DM , Feng Y , Palliser D , Weiner DB , Shankar P *et al* (2005) Antibody mediated in vivo delivery of small interfering RNAs via cell‐surface receptors. Nat Biotechnol 23: 709–717 15908939 10.1038/nbt1101

[emmm202013243-bib-0186] Starner CI , Gleason PP (2019) Spinal muscular atrophy therapies: ICER grounds the price to value conversation in facts. J Manage Care Special Pharm 25: 1306–1308 10.18553/jmcp.2019.25.12.1306 PMC10403758 31778615

[emmm202013243-bib-0187] Stein CA , Hansen JB , Lai J , Wu S , Voskresenskiy A , Hog A , Worm J , Hedtjarn M , Souleimanian N , Miller P *et al* (2010) Efficient gene silencing by delivery of locked nucleic acid antisense oligonucleotides, unassisted by transfection reagents. Nucleic Acids Res 38: e3 19854938 10.1093/nar/gkp841 PMC2800216

[emmm202013243-bib-0188] Stein H , Hausen P (1969) Enzyme from calf thymus degrading the RNA moiety of DNA‐RNA Hybrids: effect on DNA‐dependent RNA polymerase. Science 166: 393–395 5812039 10.1126/science.166.3903.393

[emmm202013243-bib-0189] Steinbacher JL , Landry CC (2014) Adsorption and release of siRNA from porous silica. Langmuir 30: 4396–4405 24087929 10.1021/la402850m PMC3997627

[emmm202013243-bib-0190] Sugo T , Terada M , Oikawa T , Miyata K , Nishimura S , Kenjo E , Ogasawara‐Shimizu M , Makita Y , Imaichi S , Murata S *et al* (2016) Development of antibody‐siRNA conjugate targeted to cardiac and skeletal muscles. J Control Release 237: 1–13 27369865 10.1016/j.jconrel.2016.06.036

[emmm202013243-bib-0191] Suhr OB , Coelho T , Buades J , Pouget J , Conceicao I , Berk J , Schmidt H , Waddington‐Cruz M , Campistol JM , Bettencourt BR *et al* (2015) Efficacy and safety of patisiran for familial amyloidotic polyneuropathy: a phase II multi‐dose study. Orphanet J Rare Dis 10: 109 26338094 10.1186/s13023-015-0326-6 PMC4559363

[emmm202013243-bib-0192] Summerton J , Weller D (1997) Morpholino antisense oligomers: design, preparation, and properties. Antisense Nucleic Acid Drug Dev 7: 187–195 9212909 10.1089/oli.1.1997.7.187

[emmm202013243-bib-0193] Szebeni J (2018) Mechanism of nanoparticle‐induced hypersensitivity in pigs: complement or not complement? Drug Discov Today 23: 487–492 29326077 10.1016/j.drudis.2018.01.025

[emmm202013243-bib-0194] Taetz S , Nafee N , Beisner J , Piotrowska K , Baldes C , Murdter TE , Huwer H , Schneider M , Schaefer UF , Klotz U *et al* (2009) The influence of chitosan content in cationic chitosan/PLGA nanoparticles on the delivery efficiency of antisense 2'‐O‐methyl‐RNA directed against telomerase in lung cancer cells. Eur J Pharma Biopharm 72: 358–369 10.1016/j.ejpb.2008.07.011 18703137

[emmm202013243-bib-0195] Takahashi K , Tanabe K , Ohnuki M , Narita M , Ichisaka T , Tomoda K , Yamanaka S (2007) Induction of pluripotent stem cells from adult human fibroblasts by defined factors. Cell 131: 861–872 18035408 10.1016/j.cell.2007.11.019

[emmm202013243-bib-0196] Takahashi K , Yamanaka S (2006) Induction of pluripotent stem cells from mouse embryonic and adult fibroblast cultures by defined factors. Cell 126: 663–676 16904174 10.1016/j.cell.2006.07.024

[emmm202013243-bib-0197] Teasdale I (2019) Stimuli‐responsive phosphorus‐based polymers. Eur J Inorg Chem 2019: 1445–1456 30983876 10.1002/ejic.201801077 PMC6446734

[emmm202013243-bib-0198] Thanki K , Zeng X , Justesen S , Tejlmann S , Falkenberg E , Van Driessche E , Morck Nielsen H , Franzyk H , Foged C (2017) Engineering of small interfering RNA‐loaded lipidoid‐poly(DL‐lactic‐co‐glycolic acid) hybrid nanoparticles for highly efficient and safe gene silencing: A quality by design‐based approach. Eur J Pharma Biopharma 120: 22–33 10.1016/j.ejpb.2017.07.014 28756280

[emmm202013243-bib-0199] Thomas GS , Cromwell WC , Ali S , Chin W , Flaim JD , Davidson M (2013) Mipomersen, an apolipoprotein B synthesis inhibitor, reduces atherogenic lipoproteins in patients with severe hypercholesterolemia at high cardiovascular risk: a randomized, double‐blind, placebo‐controlled trial. J Am Coll Cardiol 62: 2178–2184 24013058 10.1016/j.jacc.2013.07.081

[emmm202013243-bib-0200] Tuerk C , Gold L (1990) Systematic evolution of ligands by exponential enrichment: RNA ligands to bacteriophage T4 DNA polymerase. Science 249: 505–510 2200121 10.1126/science.2200121

[emmm202013243-bib-0201] Vitravene Study G (2002) A randomized controlled clinical trial of intravitreous fomivirsen for treatment of newly diagnosed peripheral cytomegalovirus retinitis in patients with AIDS. Am J Ophthalmol 133: 467–474 11931780 10.1016/s0002-9394(02)01327-2

[emmm202013243-bib-0202] Voit T , Topaloglu H , Straub V , Muntoni F , Deconinck N , Campion G , De Kimpe SJ , Eagle M , Guglieri M , Hood S *et al* (2014) Safety and efficacy of drisapersen for the treatment of Duchenne muscular dystrophy (DEMAND II): an exploratory, randomised, placebo‐controlled phase 2 study. Lancet Neurol 13: 987–996 25209738 10.1016/S1474-4422(14)70195-4

[emmm202013243-bib-0203] Vollmer J , Weeratna RD , Jurk M , Samulowitz U , McCluskie MJ , Payette P , Davis HL , Schetter C , Krieg AM (2004) Oligodeoxynucleotides lacking CpG dinucleotides mediate Toll‐like receptor 9 dependent T helper type 2 biased immune stimulation. Immunology 113: 212–223 15379982 10.1111/j.1365-2567.2004.01962.x PMC1782571

[emmm202013243-bib-0204] Wahlestedt C , Salmi P , Good L , Kela J , Johnsson T , Hokfelt T , Broberger C , Porreca F , Lai J , Ren K *et al* (2000) Potent and nontoxic antisense oligonucleotides containing locked nucleic acids. Proc Natl Acad Sci USA 97: 5633–5638 10805816 10.1073/pnas.97.10.5633 PMC25880

[emmm202013243-bib-0205] Wang S , Allen N , Prakash TP , Liang XH , Crooke ST (2019) Lipid conjugates enhance endosomal release of antisense oligonucleotides into cells. Nucleic Acid Ther 29: 245–255 31158063 10.1089/nat.2019.0794

[emmm202013243-bib-0206] Wathion N (2002) Public Statement on Vitravene (fomivirsen). https://www.ema.europa.eu/en/documents/public-statement/public-statement-vitravene-fomivirsen-withdrawal-marketing-authorisation-european-union_en.pdf

[emmm202013243-bib-0207] Watts JK , Deleavey GF , Damha MJ (2008) Chemically modified siRNA: tools and applications. Drug Discov Today 13: 842–855 18614389 10.1016/j.drudis.2008.05.007

[emmm202013243-bib-0208] Westein E , van der Meer AD , Kuijpers MJ , Frimat JP , van den Berg A , Heemskerk JW (2013) Atherosclerotic geometries exacerbate pathological thrombus formation poststenosis in a von Willebrand factor‐dependent manner. Proc Natl Acad Sci USA 110: 1357–1362 23288905 10.1073/pnas.1209905110 PMC3557050

[emmm202013243-bib-0209] Wiklander OPB , Brennan MA , Lotvall J , Breakefield XO , El Andaloussi S (2019) Advances in therapeutic applications of extracellular vesicles. Sci Transl Med 11: eaav8521 31092696 10.1126/scitranslmed.aav8521 PMC7104415

[emmm202013243-bib-0210] Willms E , Cabanas C , Mager I , Wood MJA , Vader P (2018) Extracellular vesicle heterogeneity: subpopulations, isolation techniques, and diverse functions in cancer progression. Front Immunol 9: 738 29760691 10.3389/fimmu.2018.00738 PMC5936763

[emmm202013243-bib-0211] Wong E , Goldberg T (2014) Mipomersen (kynamro): a novel antisense oligonucleotide inhibitor for the management of homozygous familial hypercholesterolemia. P T 39: 119–122 24669178 PMC3956393

[emmm202013243-bib-0600] Wu H , Lima WF , Zhang H , Fan A , Sun H , Crooke ST (2004) Determination of the role of the human RNase H1 in the pharmacology of DNA‐like antisense drugs. J Biol Chem 279: 17181–17189 14960586 10.1074/jbc.M311683200

[emmm202013243-bib-0212] Yang T , Fogarty B , LaForge B , Aziz S , Pham T , Lai L , Bai S (2017) Delivery of small interfering RNA to inhibit vascular endothelial growth factor in zebrafish using natural brain endothelia cell‐secreted exosome nanovesicles for the treatment of brain cancer. AAPS J 19: 475–486 27882487 10.1208/s12248-016-0015-y

[emmm202013243-bib-0213] Yao YD , Sun TM , Huang SY , Dou S , Lin L , Chen JN , Ruan JB , Mao CQ , Yu FY , Zeng MS *et al* (2012) Targeted delivery of PLK1‐siRNA by ScFv suppresses Her2+ breast cancer growth and metastasis. Sci Transl Med 4: 130ra148 10.1126/scitranslmed.3003601 22517885

[emmm202013243-bib-0214] Younis HS , Vickers T , Levin AA , Henry SP (2006) CpG and Non‐CpG oligodeoxynucleotides induce differential proinflammatory gene expression profiles in liver and peripheral blood leukocytes in mice. J Immunotoxicol 3: 57–68 18958686 10.1080/15476910600718236

[emmm202013243-bib-0215] Yu AM , Choi YH , Tu MJ (2020) RNA drugs and RNA targets for small molecules: principles, progress, and challenges. Pharmacol Rev 72: 862–898 32929000 10.1124/pr.120.019554 PMC7495341

[emmm202013243-bib-0216] Yu RZ , Lemonidis KM , Graham MJ , Matson JE , Crooke RM , Tribble DL , Wedel MK , Levin AA , Geary RS (2009) Cross‐species comparison of in vivo PK/PD relationships for second‐generation antisense oligonucleotides targeting apolipoprotein B‐100. Biochem Pharmacol 77: 910–919 19056355 10.1016/j.bcp.2008.11.005

[emmm202013243-bib-0217] Zamecnik PC , Stephenson ML (1978) Inhibition of Rous sarcoma virus replication and cell transformation by a specific oligodeoxynucleotide. Proc Natl Acad Sci USA 75: 280–284 75545 10.1073/pnas.75.1.280 PMC411230

[emmm202013243-bib-0218] Zhang A , Uaesoontrachoon K , Shaughnessy C , Das JR , Rayavarapu S , Brown KJ , Ray PE , Nagaraju K , van den Anker JN , Hoffman EP *et al* (2015) The use of urinary and kidney SILAM proteomics to monitor kidney response to high dose morpholino oligonucleotides in the mdx mouse. Toxicol Rep 2: 838–849 26213685 10.1016/j.toxrep.2015.05.008 PMC4512206

